# Conserved components of the macroautophagy machinery in *Caenorhabditis elegans*

**DOI:** 10.1093/genetics/iyaf007

**Published:** 2025-04-04

**Authors:** Hong Zhang, Alicia Meléndez

**Affiliations:** National Laboratory of Biomacromolecules, New Cornerstone Science Laboratory, Institute of Biophysics, Chinese Academy of Sciences, Beijing 100101, P.R. China; College of Life Sciences, University of Chinese Academy of Sciences, Beijing 100049, P.R. China; Department of Biology, Queens College, City University of New York, Flushing, NY 11367, USA; Molecular, Cellular and Developmental Biology and Biochemistry Ph.D. Programs, The Graduate Center of the City University of New York, New York, NY 10016, USA

**Keywords:** WormBook, *C. elegans*, autophagy, mitophagy, lysosome, P granules, development, longevity, dietary restriction, aggrephagy, lysophagy, lipophagy, xenophagy, hormesis

## Abstract

Macroautophagy involves the sequestration of cytoplasmic contents in a double-membrane autophagosome and its subsequent delivery to lysosomes for degradation and recycling. In *Caenorhabditis elegans,* autophagy participates in diverse processes such as stress resistance, cell fate specification, tissue remodeling, aging, and adaptive immunity. Genetic screens in *C. elegans* have identified a set of metazoan-specific autophagy genes that form the basis for our molecular understanding of steps unique to the autophagy pathway in multicellular organisms. Suppressor screens have uncovered multiple mechanisms that modulate autophagy activity under physiological conditions. *C. elegans* also provides a model to investigate how autophagy activity is coordinately controlled at an organismal level. In this chapter, we will discuss the molecular machinery, regulation, and physiological functions of autophagy, and also methods utilized for monitoring autophagy during *C. elegans* development.

## General background of autophagy

Autophagy is an evolutionarily conserved catabolic process, essential to maintain cellular homeostasis and normal physiology. DeDuve first coined the term “autophagy” to describe the phenomenon in which autophagosomes fuse with lysosomes, resulting in the degradation of its contents (DeDuve 1963, 1964). Several decades later, the discovery of autophagy-related (*atg* genes) provided a framework to study the core molecular machinery that controls the process of autophagy (Klionsky *et al.* 2003; Ohsumi 2014). This review will discuss the proteins involved in autophagy and the role of this process in *Caenorhabditis elegans* development, and stress. We will describe how studies in *C. elegans* have advanced our molecular understanding of the process and its regulation in multicellular organisms. We will also discuss how specific cargos, such as protein aggregates and paternal organelles are selectively targeted for autophagic degradation. The physiological function of autophagy during *C. elegans* development has been extensively reviewed recently (Yang and Zhang 2014; Zhang and Baehrecke 2015; Hansen *et al.* 2018; Palmisano and Meléndez 2019), and thus we will present only the most recent advances.

There are several forms of autophagy, and they are distinguished based on the mechanism by which the cargo is sequestrated: macroautophagy, microautophagy, and chaperone-mediated autophagy (CMA). CMA involves the selection and direct delivery of individual cytosolic proteins, on the basis of the KFERQ-like motif in their sequence (Kaushik and Cuervo 2018; Bourdenx *et al.* 2021). Microautophagy consists of the involution by pinocytosis of cargo directly into the lysosome prior to degradation (Wang and Klionsky 2003; Oku and Sakai 2018; Schuck 2020; Kuchitsu and Taguchi 2023). Early studies of microautophagy in rat liver found that microautophagy is the main autophagic response under starvation (Mortimore *et al.* 1983, 1988; de Waal *et al.* 1986). A form of microautophagy where cytosolic proteins are delivered to the endosome is referred to as endosomal Microautophagy (eMI) (Sahu *et al.* 2011). eMI can also be a selective process mediated by the heat shock protein HSPA8, where similar to CMA, the chaperone protein recognizes KFERQ-like motifs. However, very little is known about these processes in *C. elegans*, even though many proteins contain the KFERQ-like motifs (Meléndez A, personal communication). Thus, further studies are warranted in *C. elegans* to better understand the processes involved and the level of cross talk that exists between the different degradation pathways.

**Fig. 1.**
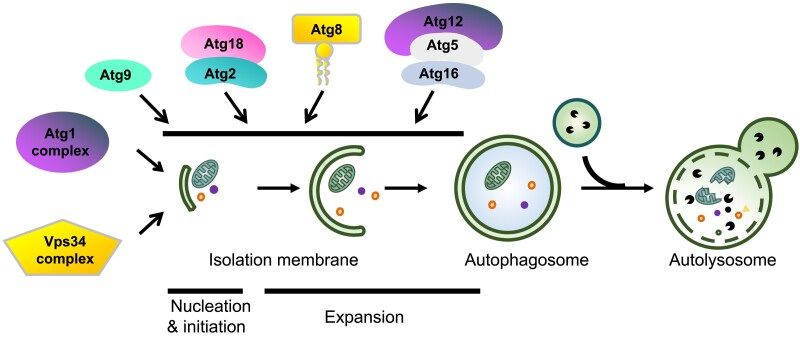
Overview of the macroautophagy process in *C. elegans*. The process of autophagy proceeds through discrete steps beginning with initiation, membrane nucleation, IM, or phagophore formation, IM/phagophore elongation, fusion with the lysosome to form an autolysosome, degradation and finally, recycling of the autophagosomal cargo.

Macroautophagy (hereafter autophagy) involves the engulfment of cytoplasmic contents in a double-membrane autophagosome and its delivery to the vacuole (in yeast or plant) or lysosomes (in multicellular animals) for degradation (Lamb *et al.* 2013; Feng *et al.* 2014; Zhao and Zhang 2019a; Nakatogawa 2020; Zhao *et al.* 2021) (Fig. 1). The formation of the autophagosome can be dissected into several membrane remodeling steps, including initiation and nucleation of an isolation membrane (IM), also known as the phagophore, IM expansion, and closure, and finally, the fusion of the autophagosome with the lysosome for degradation (Lamb *et al.* 2013; Feng *et al.* 2014) (Fig. 1). Autophagy acts as a mechanism for coping with metabolic stresses and also has a scavenging function by selectively removing damaged/superfluous organelles and protein aggregates formed by misfolded/mutant proteins to maintain cellular homeostasis (Stolz *et al.* 2014; Anding and Baehrecke 2017; Lamark and Johansen 2021). Dysfunction of autophagy has been causatively linked to the pathogenesis of a variety of human diseases such as tumorigenesis, neurodegeneration, and immune diseases (Mizushima *et al.* 2008; Deretic and Levine 2018; Levine and Kroemer 2019; Zhao and Zhang 2019b; Yang and Klionsky 2020).

## Autophagic machinery for autophagosome formation based on yeast genetic screens

**Fig. 2.**
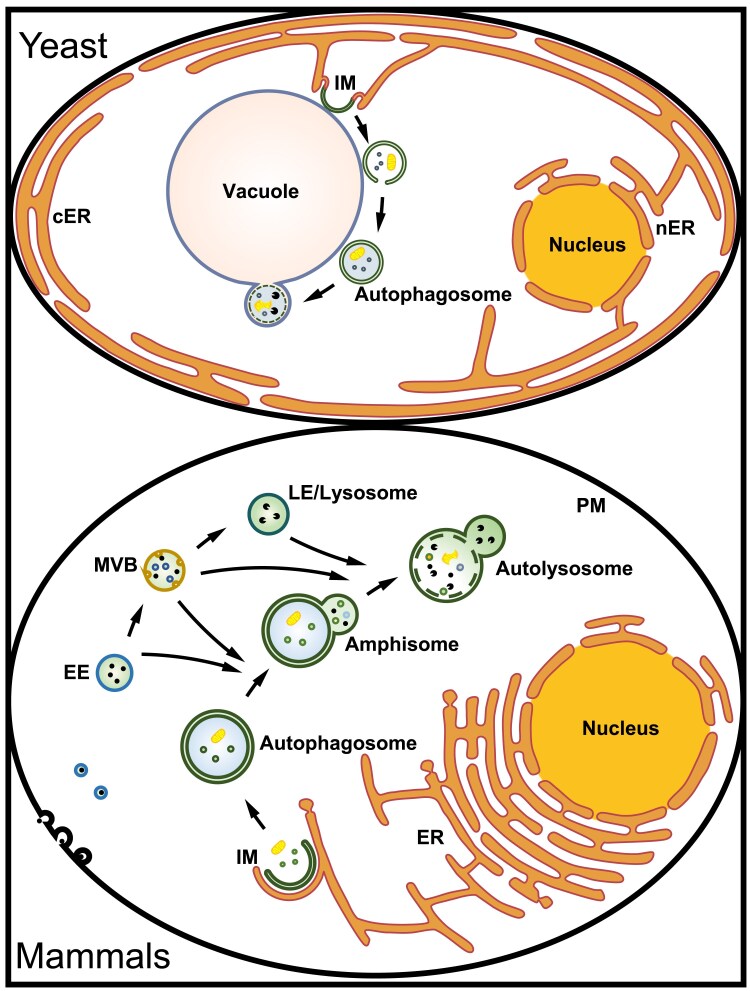
The differences in the autophagy pathway in yeast and multicellular animals. In yeast, autophagosomes are generated at a single site located on the vacuole, known as the PAS. The ER is enriched in the perinuclear and cortical regions. The leading edges of the growing IM form contacts with the ER. Nascent autophagosomes directly fuse with the vacuole. cER, cortical ER; nER, perinuclear ER; IM, isolation membrane. In mammalian cells, the ER is widely distributed in the cytosol. IMs are simultaneously formed at multiple sites on the ER. The expanding IMs form extensive and highly dynamic contacts with the ER. Nascent autophagosomes fuse with vesicles originating from endolysosomal compartments to form intermediates called amphisomes and eventually degradative autolysosomes, a process known as autophagosome maturation. EE, early endosome; LE, late endosome; MVB, multivesicular body; PM, plasma membrane.

**Fig. 3.**
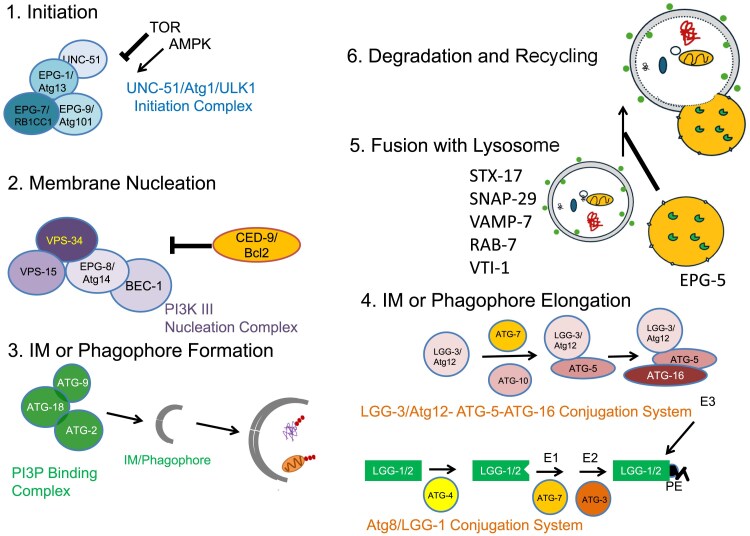
The function of ATG proteins at different steps of autophagosome formation. ATG proteins act at different steps of autophagosome formation. See the main text for the detailed function of each ATG protein. Steps are executed by distinct complexes of proteins: (1) Initiation is executed by the UNC-51/Atg1/ULK1 initiation complex; (2) Membrane nucleation is executed by the class III PtdIns 3-kinase nucleation complex; (3) IM or phagophore formation requires the PtdIns3P-binding complex; (4) Elongation of the IM or phagophore requires the LGG-3/Atg12 conjugation system and the Atg8/LGG-1 or LGG-2 conjugation system; (5) The fusion of the autophagosome with a lysosome to form an autolysosome is mediated by a SNARE complex, which consists of STX-17/Syntaxin 17 (a t-SNARE protein) on autophagosomes, SNAP-29 (a SNARE protein), and the endosomal/lysosomal R-SNARE VAMP-7 (Itakura *et al*. 2008). This fusion process is regulated by several factors, including the late endosomal/lysosomal-localized protein PLEKHM1 and TECPR1 (which interacts with PtdIns(3)P and the Atg12-Atg5 conjugate) (Chen *et al*. 2013; McEwan *et al*. 2015). For all proteins, we indicate the *C. elegans* protein first and the yeast ortholog and the mammalian ortholog. As part of the conjugation system LGG-1 or LGG-2 is cleaved by ATG-4.1 or ATG-4.2 and conjugated to PE on the autophagosome membrane, which incorporates pre-autophagosomal and autophagosomal membranes.

Genetic screens in yeast have identified a set of autophagy-related (*ATG*) genes essential for autophagosome biogenesis (Table 1 and Fig. 2). These screens are mainly based on defects in the accumulation of autophagic bodies (ABs: cargo-containing unruptured inner membranes) in the vacuole upon starvation, or on impaired delivery of the precursor of aminopeptidase I (prApeI) into the vacuole by the Cvt pathway (Takeshige *et al.* 1992; Tsukada and Ohsumi 1993; Baba *et al.* 1994; Harding *et al.* 1995, 1996). Atg proteins are recruited in a hierarchical order to the pre-autophagosomal structure (PAS) and act at discrete steps of autophagosome formation (Figs. 1 and 3). In brief, upon autophagy induction, the serine/threonine kinase Atg1 kinase complex (composed of Atg1, Atg13, and Atg17) acts at the most upstream stage to organize the autophagosome formation site (Noda and Fujioka 2015). Starvation-triggered dephosphorylation of Atg13 leads to the formation of the Atg1 complex that further undergoes liquid–liquid phase separation (LLPS) (Kamada *et al.* 2000; Fujioka *et al.* 2014, 2020; Yamamoto *et al.* 2016; Memisoglu and Haber 2019; Fujioka *et al.* 2020). The resultant puncta (also known as condensates) are tethered to the vacuolar membrane via interaction with the vacuolar membrane protein Vac8 (Hollenstein *et al.* 2019; Fujioka *et al.* 2020). The class III phosphatidylinositol 3-kinase (PI(3)K) Vps34 complex (composed of Vps34, Vps15, Atg6, and Atg14) is then recruited to the early PAS for initiation of autophagosome formation (Suzuki *et al.* 2007). During IM expansion, also referred to as phagophore expansion, the Atg2–Atg18 complex locates to the extremities of the IM, whose spatial restriction requires the multispanning membrane protein Atg9. The Atg2–Atg18 complex tethers the ER to the leading edges of the growing IMs by simultaneously binding to the ER and the IM (Gómez-Sánchez *et al.* 2018; Kotani *et al.* 2018). Atg2 possesses lipid-transfer activity and the Atg2–Atg18 complex directly transfers phospholipids from the ER to the IM (Maeda *et al.* 2019; Osawa *et al.* 2019; Valverde *et al.* 2019). Phospholipids transported by Atg2 are translocated from the cytoplasmic to the luminal leaflet by the lipid scramblase Atg9, thus driving IM expansion (Osawa *et al.* 2019). Atg9, which traffics between mobile Atg9-positive vesicles and the PAS, also plays a role in organizing the PAS. Two ubiquitin-like conjugation systems function at multiple steps of autophagosome formation, including IM expansion, shaping, and closure (Mizushima and Komatsu 2011; Mizushima 2020; Nakatogawa 2020). Newly synthesized ubiquitin-like protein Atg8 is cleaved by the cysteine protease Atg4 to expose its C-terminal glycine, which is then conjugated to phosphatidylethanolamine (PE) through the sequential actions of the E1-like activating enzyme Atg7 and the E2-like conjugating enzyme Atg3. The actions of Atg7 and the E2-like enzyme Atg10 mediate the conjugation of the ubiquitin-like protein Atg12 to Atg5. The Atg5–Atg12 conjugate further interacts with Atg16, which in turn acts as an E3-like enzyme to facilitate Atg8-PE conjugation (Mizushima 2020). Autophagosome sealing occurs concomitantly with disassociation of Atg proteins from the IM, which requires clearance of PI(3)P. Depletion of the myotubularin family PI(3)P phosphatase Ymr1 causes persistence of Atg proteins on autophagosomal membranes and inhibits subsequent fusion with vacuoles (Cebollero *et al.* 2012). ESCRT complex-mediated membrane abscission drives autophagosome closure (Takahashi *et al.* 2018, 2019; Zhen *et al.* 2020). Subsequent fusion of autophagosomes with the vacuole is mediated by the SNARE complex composed of Ykt6 on the autophagosome and Vam3, Vam7, and Vti1 on the vacuole (Bas *et al.* 2018). The vacuolar-localized phospholipase Atg15, a phospholipase B of broad substrate specificity, acts to disintegrate AB membranes (Kagohashi *et al.* 2023; Watanabe *et al.* 2023).

**Table 1.** Autophagy-related genes and autophagy regulators in yeast, *C. elegans,* and mammals.

	Yeast *ATG* gene	*C. elegans ATG* homolog	Mammalian *ATG* homolog
Atg1/ULK complex	*atg1*	*unc-51*	*ULK1/ULK2*
	*atg13*	*epg-1*	*ATG13*
	*atg17*	*epg-7*	*FIP200/RB1CC1*
		*epg-9*	*ATG101*
Class III (PI(3)K) Vps34 complex	*atg6/vps30*	*bec-1*	*BECN1*
	*vps34*	*vps-34*	*PIK3C3/VPS34*
	*vps15*	*vps-15*	*PIK3R4/VPS15*
	*atg14*	*epg-8*	*ATG14L*
PI3P binding complex	*atg18*	*atg-18, epg-6*	*WIPI1, WIPI2, WIPI3, WIPI4*
	*atg2*	*atg-2*	*ATG2A, ATG2B*
	*atg9*	*atg-9*	*ATG9A, ATG9B*
Atg8 ubiquitin-like conjugation system	*atg4*	*atg-4.1, atg-4.2*	*ATG4A, ATG4B, ATG4C, ATG4D*
	*atg3*	*atg-3*	*ATG3*
	*atg7*	*atg-7*	*ATG7*
	*atg8*	*lgg-1/lgg-2*	*LC3A, LC3B, LC3C, GABARAP, GABARAPL1, GABARAPL2*
Atg12 ubiquitin-like conjugation system	*atg5*	*atg-5*	*ATG5*
	*atg7*	*atg-7*	*ATG7*
	*atg10*	*atg-10*	*ATG10*
	*atg12*	*lgg-3*	*ATG12*
	*atg16*	*atg-16.1, atg-16.2*	*ATG16L1, ATG16L2*
Autophagosome maturation		*mtm-3*	*MTMR3*
		*epg-5*	*mEPG5*
	*vam3*	*syx-17*	*STX17*
		*snap-29*	*SNAP29*
		*ogt-1*	*OGT*
		*vamp-7*	*VAMP8*
	*ypt7*	*rab-7*	*RAB7A*
	*vti1*	*vti-1*	*VTI1a, VT1b*
		*cup-14*	*PLEKHM1*
Autophagy-related		*epg-2*	
		*epg-3*	*VMP1*
		*epg-4*	*EI24*
		*lpla-2*	*PLA2G15*
		*sepa-1*	
		*rub-1*	*RUBCN/RUBICON*
	*kog1*	*daf-15*	*RPTOR*
	*tsc11*	*rict-1*	*RICTOR*
	*rhb1*	*rheb-1*	*RHEB*
		*ced-9*	*BCL2*
	*vps4*	*vps-4*	*VPS4A, VPS4B, SKD1*
	*ypt31, ypt32*	*rab-11.1, rab-11.2*	*RAB-11A*
	*ssa1-4*	*hsp-70*	*HSPA8/HSC70*
	*hsc82, hsp82*	*daf-21*	*HSP90AA1*
	*vps38*	*T23G11.7, Y34BA.2*	*UVRAG*
Regulate autophagy gene expression	*TOR1, TOR2*	*let-363/TOR*	*MTOR*
		*hlh-30*	*TFEB, TFE3*
		*daf-16*	*FOXO*
		*pha-4*	*FOXA*
	*impk*	*impk-1*	*IMPK*
Selective autophagy receptor for ubiquitinated cargo		*sqst-1*	*SQST-1/p62*
Selective autophagy– mitophagy		*pdr-1*	*PRKN*
		*pink-1*	*PINK1*
		*dct-1*	*BNIP3*
Selective autophagy– allophagy		*fundc-1*	*FUNDC1A*
	*phb2*	*phb-2*	*PHB2*
		*allo-1a, allo-1b*	
	*cak1*	*ikke-1*	*TBK1*
HOPS complex, for vesicle fusion	*vps11*	*vps-11*	*VPS11*
	*vps18*	*vps-18*	*VPS18*
	*vam6/vps39*	*vps-39*	*VPS39*
	*vam2/vps41*	*vps-41*	*VPS41*
	*vps16*	*vps-16*	*VPS16*
	*vps33*	*vps-33.1, vps-33.2*	*VPS33A, VPS33B*

In mammalian cells, Atg proteins may be differentially employed due to the assembly of autophagosomes on the ER (Table 1 and Fig. 2). For example, the composition and regulatory mode of the Atg1 complex differ substantially between yeast and mammals. The mammalian counterpart of the Atg1 complex consists of ULK1, FIP200, ATG13 (a highly divergent homolog of Atg13), and ATG101 (Mizushima 2010; Feng *et al.* 2014). Despite its high sequence diversity, FIP200 may be a functional counterpart of yeast Atg17. ATG101 is absent in budding yeast (Mizushima 2010; Liang *et al.* 2012). Upon starvation, the ULK1/ATG13/FIP200 complex forms ER-associated puncta (Itakura and Mizushima 2010). Autophagy stimuli elicit Ca^2+^ transients on the ER outer surface that further drive LLPS of the ULK1/FIP200 complex (Zheng *et al.* 2023). The resultant FIP200 condensates associate with the ER via interaction with VAPA/B and ATL2/3 and organize into autophagosome formation sites (Zheng *et al.* 2022). The VPS34 complex, consisting of VPS34, Atg6/Beclin 1, VPS15 and a highly divergent Atg14 homolog, Atg14L, is then recruited to generate PI(3)P (Feng *et al.* 2014). ATG9 vesicles also contribute to the organization of autophagosome initiation sites, while their function in autophagosome formation remains elusive (Yamamoto *et al.* 2012). IMs forms extensive and highly dynamic contacts with the ER, which may allow lipid transport from the ER to IMs at the contact sites (Hayashi-Nishino *et al.* 2009; Yla-Anttila *et al.* 2009). The ER-IM contact is tethered by the interaction of the integral ER proteins VAPA, VAPB, ATL2, and ATL3 with components of the FIP200–ULK1 complex, and also interactions among ATG proteins and PI(3)P located at the ER and IMs (Zhao *et al.* 2017, 2018; Liu *et al.* 2021b).

Mammalian cells possess multiple homologs of the same yeast Atg proteins, which show functional redundancy, as well as functional divergence. This confers another layer of complexity. Mammalian cells contain six ATG8 homologs, three in the LC3 subfamily (LC3A, LC3B, and LC3C) and three in the GABARAP subfamily (GABARAP, GABARAPL1, and GABARAPL2). In cells depleted of all six ATG8s, autophagosome closure is severely delayed and the autophagosomes are smaller (Nguyen *et al.* 2016). There are four mammalian homologs of yeast Atg18, WIPI1-4, with distinct function in autophagy (Proikas-Cezanne *et al.* 2015). WIPI2 directly interacts with ATG16 and defines the action site for the ATG16-ATG12-ATG5 complex, and also contributes to the formation of ER-IM contacts (Dooley *et al.* 2014; Zhao *et al.* 2017). WIPI4 shows stronger interaction with ATG2A/B than WIPI1 and WIPI2 (Zheng *et al.* 2017). The ESCRT complex drives the closure of IMs into autophagosomes (Yu and Melia 2017; Takahashi *et al.* 2018). Fusion of autophagosomes with late endosomes/lysosomes is mediated by two partially functionally redundant SNARE complexes formed by autophagosomal membrane-localized STX17 (Qa) and SNAP29 (Qbc) and late endosomal/lysosomal-localized VAMP8, or by the autophagosomal YKT6, SNAP29, and STX7 (Matsui *et al.* 2018).

In *C. elegans*, homologs of *Atg1-10*, *Atg12*, *Atg16,* and *Atg18* are conserved and are also essential for autophagy (Table 1; Figs. 1 and 3). *C. elegans* has two homologs of yeast *Atg4 (C. elegansatg-4.1* and *atg-4.2)*, *Atg8 (C. eleganslgg-1* and *lgg-2),* and *Atg16* (*C. elegansatg-16.1 and atg-16.2*) (Wu *et al.* 2012, 2015; Zhang *et al.* 2013). *atg-16.2* mutants exhibit a stronger autophagic defect (i.e. accumulation of protein aggregates and formation of LGG-1 puncta) than *atg-16.1* mutants. *atg-16.2; atg-16.1* double mutants display a much more severe defect than either single mutant (Zhang *et al.* 2013). Loss of *atg-4.1* activity causes defective degradation of a variety of protein aggregates, whereas *atg-4.2* mutants show no defects (Wu *et al.* 2012). *atg-4.1* and *atg-4.2* function redundantly in LGG-1 processing. ATG-4.1 preferentially cleaves the soluble pro-form of LGG-1 for its conjugation onto the autophagosome, while ATG-4.2 preferentially cleaves autophagic membrane-bound to LGG-1/LGG-2 (Wu *et al.* 2012; Hill *et al.* 2019). Loss of function of *atg-4.2* causes the accumulation of immature autophagosomes in neurons (Hill *et al.* 2019). LGG-1 and LGG-2 in *C. elegans*, which belong to the GABARAP and LC3 families, respectively, act differentially in autophagy (Wu *et al.* 2015). LGG-1 is closer to the yeast Atg8 and could complement the loss of viability of *ATG8*-disrupted yeast under nutrient starvation, whereas LGG-2 failed to complement in parallel experiments (Alberti *et al.* 2010). LGG-1 is essential for the degradation of various protein aggregates, while LGG-2 has cargo-specific and developmental stage-specific roles. LGG-1 depletion blocks autophagosome formation, while loss of LGG-2 causes the formation of smaller autophagosomes (Wu *et al.* 2015). LGG-2 also participates in maturation of paternal organelle-containing autophagosomes during embryogenesis via direct interaction with the HOPS complex subunit-VPS-39 (see below) (Manil-Ségalen *et al.* 2014; Djeddi *et al.* 2015). Loss of function of the *C. elegans* myotubularin family PI(3)P phosphatase MTM-3 causes persistent association of ATG-18 and impairs autophagosome maturation (Wu *et al.* 2014). The STX-17-SNAP29-VAMP-7 complex appears to mediate the fusion of autophagosomes with late endosomes/lysosomes (Guo *et al.* 2014b).

## Identifying novel components of the autophagy pathway in *C. elegans*

**Fig. 4.**
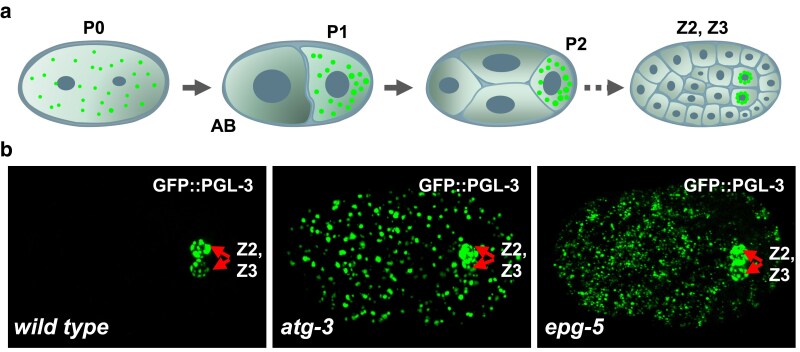
Accumulation of PGL granules in somatic cells in autophagy gene mutant embryos. a) Schematic illustration of localization of the oocyte-derived P granules during early asymmetric divisions generating somatic and germline cells in *C. elegans* embryos (*left*). P granules are indicated as small green dots. b) Three-dimensional projection images of the distribution of GFP::PGL-3-labeled granules in wild-type embryos, *atg-3* mutant embryos and *epg-5* mutant embryos. The Z2 and Z3 germ precursor cells are indicated by red arrows.

**Fig. 5.**
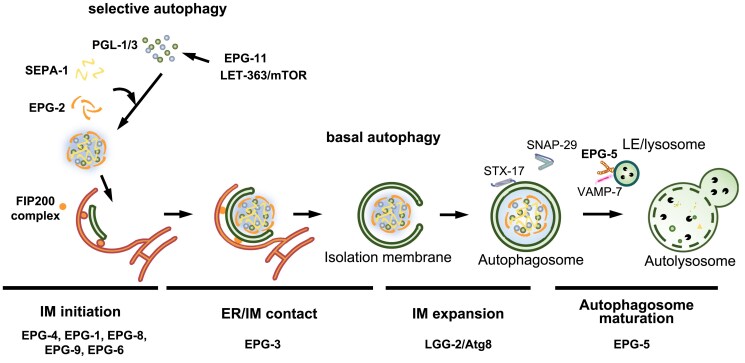
The role of *epg* genes identified from *C. elegans* genetic screens in the aggrephagy pathway. Schematic illustration of the role and the hierarchical order of autophagy proteins in the aggrephagy pathway in *C. elegans*. LE, late endosome. EPG-11 and LET-363/mTOR mediate posttranslational modification (PTM) of PGL-1 and PGL-3. EPG-11 encodes the PRMT1 homolog and mediates arginine methylation in the RGG (Arg–Gly–Gly) domains of PGL-1 and PGL-3 (Li *et al*. 2012). EPG-11- and LET-363-mediated PTMs modulate phase separation of PGL-1 and PGL-3 (Zhang *et al*. 2018). See the main text for the detailed function of each EPG protein.

Forward genetic screens in *C. elegans* have yielded conserved components originally identified in yeast screens, underscoring the conservation of basic mechanisms, as well as novel genes that lack yeast homologs but are conserved in mammals, underscoring the value of a metazoan genetic system for studying autophagy. Genetic screens for genes that are essential for degradation of protein substrates during *C. elegans* embryogenesis (see below, Fig. 4) identified highly conserved *atg* genes. *C. elegans* also contains highly divergent functional homologs of Atg13 and Atg14, encoded by *epg-1* (*e*ctopic *P*GL *g*ranules) and *epg-8*, respectively (Table 1 and Fig. 5) (Tian *et al.* 2009, 2010a, 2010b; Lu *et al.* 2011; Yang and Zhang 2011; Liang *et al.* 2012; Wu *et al.* 2015). *epg-9* encodes the mammalian ATG101 homolog (Liang *et al.* 2012). EPG-7 displays similarity to FIP200, but it is not essential for autophagy (Lin *et al.* 2013). Genetic screens also identified several genes, including *epg-3*, -*4,* and -5, whose homologs are found in mammals but are absent in yeast (Table 1 and Fig. 4) (Tian *et al.* 2010a). *epg-3* and *epg-4* encode ER transmembrane proteins and act at an early step of autophagosome formation (Fig. 5) (Zhao *et al.* 2017; Zheng *et al.* 2022). EI24/EPG4 (mammalian homolog of EPG-4) controls the frequency, amplitude, and duration of ER Ca^2+^ transients. In *EI24* KO cells, persistent ER Ca^2+^ transients cause accumulation of FIP200 puncta and LC3 structures that are not functional (Zheng *et al.* 2022). VMP1/EPG3 (mammalian homolog of EPG-3) modulates the disassembly of the ER-IM contacts (Fig. 5). *VMP1* depletion causes stable association of IMs with the ER, so that IMs fail to proceed into closed autophagosomes (Zhao *et al.* 2017). *C. elegans* genetic screens also identified EPG-6, which like ATG-18, also contains WD40 repeat PtdIns(3)P-binding domains. EPG-6, but not ATG-18, binds to ATG-2 (Fig. 5) (Lu *et al.* 2011). Genetic epistasis analysis placed ATG-18 upstream of EPG-6 in the autophagy pathway (Lu *et al.* 2011). The molecular mechanism of ATG-18 in autophagosome formation has yet to be determined.

Membrane fusion is promoted by a family of tethering factors that act at the initial capture of transport vesicles and accelerate the assembly of SNARE complexes for fusion with target membranes. Tethering factors are targeted to cognate membranes by binding to activated forms of small GTPases, phospholipids, and SNARE proteins (Jahn and Scheller 2006; Yu and Hughson 2010; Langemeyer *et al.* 2018). *epg-5*, which is essential for the formation of degradative autolysosomes, encodes a tether to confer the fusion specificity of autophagosomes with late endosomes/lysosomes (Tian *et al.* 2010a; Wang *et al.* 2016). Loss of mammalian EPG5 causes accumulation of autophagosomes and nondegradative autolysosomes due to nonspecific fusion of autophagosomes with other endocytic vesicles such as recycling endosomes (Wang *et al.* 2016). EPG5 is a RAB7 effector and localizes on late endosomes/lysosomes. EPG5 recognizes autophagosomes by directly binding to LC3 associated with the outer autophagosomal membrane. EPG5 stabilizes and facilitates the assembly of STX17-SNAP29-VAMP8 SNARE complexes (Wang *et al.* 2016). Other tethering factors such as PLEKHM1 and the HOPS complex also ensure fusion fidelity and efficiency during autophagosome maturation (Jiang *et al.* 2014; Takáts *et al.* 2014; McEwan *et al.* 2015).

The lysosomal-localized LPLA-2 (*C. elegans* homolog of the lysosomal phospholipase A2 family protein PLA2G15) is essential for degradation of autophagic membranous contents (Li *et al.* 2022). Loss of *lpla-2* activity causes accumulation of enlarged lysosomes, containing autophagic vesicles, undigested membranes, and autophagy cargos. Knockdown of PLA2G15 in mammalian cells also causes accumulation of enlarged lysosomes with intact autophagic vesicles (Li *et al.* 2022). In summary, characterization of autophagy genes identified from *C. elegans* facilitates our molecular understanding of steps unique to autophagy in multicellular organisms.

## Regulation of autophagy activity under physiological conditions

Studies of yeast and cultured cells have identified numerous factors that integrate various stressors into the autophagic machinery to control autophagy activity (Russell *et al.* 2014). The Atg1/ULK complex and the VPS34 complex are the two most extensively studied nodes for integrating the status of nutrients, cellular energy, and various signaling pathways with autophagy regulation (Russell *et al.* 2014). mTORC1- and AMPK-mediated phosphorylation of components of the ULK1/FIP200/ATG13 complex regulate the kinase activity of ULK1 (Russell *et al.* 2014). In *C. elegans*, inactivation of *let-363/mTOR* and activated *aak-2/AMPK* also elevate autophagy activity (Lin *et al.* 2013; Zhang *et al.* 2019). Suppressor screens in *C. elegans* have revealed multiple mechanisms that modulate autophagy activity under physiological conditions.

## Transcriptional control of autophagy genes

RNAi screens for gene inactivation that suppresses the accumulation of aggregates of the autophagy substrate SQST-1/p62 in *rpl-43* mutants, which exhibit impaired function of ribosomal protein RPL-43, revealed that autophagy genes can be transcriptionally induced by inactivation of the TGF-β Sma/Mad pathway and the *lin-35* SynMuv B pathway, and by the transcription factor PHA-4/FOXA, the XBP-1-mediated ER stress pathway, and the mitochondrial stress pathway mediated by the bZip transcription factor ATFS-1 (Guo *et al.* 2014a). Several other transcription factors are also involved in modulating the expression level of autophagy and lysosome genes under different conditions (see the “*Autophagy in Longevity–Regulation of autophagy for longevity” section, Table 1*). Thus, transcriptional regulation of autophagy genes is a widely employed mechanism to control autophagy activity by various developmental signals and stresses.

## Modulation of autophagosome maturation

Accumulation of nondegradative autolysosomes is a key feature of disorders such as neurodegenerative diseases (Settembre *et al.* 2008; Vergarajauregui *et al.* 2008; Zhao *et al.* 2021). Impaired autophagosome maturation caused by autophagy gene deficiency may be attenuated by promoting the activity of a partially redundant mechanism in this process (Zheng *et al.* 2022). Genetic screens for suppressors of the autophagy defect in *epg-5* mutants revealed multiple mechanisms to modulate autophagosome maturation.

### ogt-1

Mutations in *ogt-1*, encoding *O*-linked -*N*-acetylglucosamine (*O*-GlcNAc) transferase, were identified to suppress the accumulation of SQST-1/p62 aggregates in *epg-5(tm3425)* null mutant embryos (Guo *et al.* 2014a, 2014b). OGT mediates *O*-GlcNAcylation of SNAP-29 at multiple serine/threonine residues (Ser70, Ser134, Thr143, and Ser249). A transgene expressing *O*-GlcNAcylation-defective SNAP29 promotes autophagic flux in *C. elegans*. *OGT* knockdown also elevates autophagic flux in mammalian cells by facilitating autophagosome maturation. SNAP29 is *O*-GlcNAcylated at Ser2, Ser61, Thr130, and Ser153. *O*-GlcNAc modification attenuates the interaction of SNAP29 with STX17 and VAMP8, while expression of *O*-GlcNAcylation-defective SNAP29 facilitates the formation of SNAP29-containing SNARE complexes (Guo *et al.* 2014b). Levels of UDP-GlcNAc, the donor for *O*-GlcNAc addition, are responsive to the availability of glucose, fatty acids, uridine, and glutamine (Slawson *et al.* 2010). The levels of UDP-GlcNAc and *O*-GlcNAc-modified SNAP29 are dramatically reduced by nutrient starvation and in starved worms. Thus, SNAP29 *O*-GlcNAcylation serves as a cellular mechanism for integrating nutrient availability with autophagosome maturation (Guo *et al.* 2014b).

### susr-2

Loss-of-function mutation in *susr-2(bp1356)*, which encodes an ER transmembrane protein, also suppresses the accumulation of SQST-1/p62::GFP aggregates in *epg-5* null mutants (Miao *et al.* 2020). *susr-2(bp1356)* promotes delivery and/or maturation of lysosomal hydrolytic enzymes. *SUSR2/TMEM39A* knockdown also suppresses the autophagy defect caused by EPG5 deficiency in mammalian cells. SUSR2 acts as an adaptor protein for efficient export of the ER-localized PI(4)P phosphatase SAC1 from the ER, which in turn regulates the spatial distribution and levels of PI(4)P. Depletion of *SUSR-2* elevates late endosomal/lysosomal PI(4)P levels, facilitating recruitment of the HOPS complex to promote assembly of the SNARE complex for autophagosome maturation (Miao *et al.* 2020).

### ipmk-1

Loss-of-function mutation in *ipmk-1(bp1075)*, which encodes the ortholog of inositol polyphosphate multikinase (IPMK), suppresses the accumulation of SQST-1/p62 aggregates in *epg-5* mutants (Chen *et al.* 2020). IPMK-1 regulates autophagy independent of its catalytic activity which generates inositol tetrakis- and pentakisphosphates (e.g. IP4 and IP5) (Chen *et al.* 2020). *ipmk-1(bp1075)* mutants contain more lysosomes than control animals and show enhanced delivery and/or maturation of lysosomal hydrolytic enzymes (Chen *et al.* 2020). In mammalian cells, *IPMK* knockout elevates lysosomal function and biogenesis and suppresses the autophagy defect caused by EPG5 deficiency (Chen *et al.* 2020). The helix–loop–helix protein TFEB is a well-characterized transcription factor that activates genes essential for the autophagy-lysosomal pathway (Settembre *et al.* 2008). Nuclear-localized IPMK negatively regulates the transcriptional activity of TFEB by acting as a chaperone to inhibit LLPS of TFEB, which is involved in gene transcription (Chen *et al.* 2020).

### *rbg-1* and *rbg-2*

Upon degradation of the sequestrated materials, lysosomes are regenerated from autolysosomes to sustain autophagic flux (Yu *et al.* 2010). Dynamics movement of RAB-7 is essential for lysosomal biogenesis. RAB-7 release may promote the transport of lysosomal enzymes and/or lysosomal membrane proteins (Wang and Zhang 2019; Zhao and Zhang 2019a). In *epg-5* mutants, the mobility of late endosome/lysosome-associated RAB-7 is reduced, which may lead to recruitment of effectors such as HOPS to promote abnormal fusion and also inhibit membrane remodeling. Mutations in *rbg-1* and *rbg-2*, whose encoded proteins form a complex, ameliorate the autophagy defect in *epg-5* null mutants (Wang and Zhang 2019). The RBG-1/RBG-2 complex modulates the dynamics of membrane-associated RAB-7 to regulate lysosomal biogenesis and function. Expression of the GDP-bound form of RAB-7 also promotes lysosomal biogenesis and suppresses the autophagy defect in *epg-5* mutants (Wang and Zhang 2019).

Taken together, the above-mentioned studies show that facilitating specific fusion of autophagosomes with late endosomes/lysosomes or remodeling of stalled nondegradative autolysosomes can attenuate the autophagy defect caused by *EPG5* deficiency (Guo *et al.* 2014b; Wang and Zhang 2019; Chen *et al.* 2020; Miao *et al.* 2020). Vici syndrome is a progressive multisystem disease caused by mutations in *EPG5*. Patient tissues, as in *epg-5* mutant animals (Fig. 4, a and b), accumulate nondegradative autolysosomes (Cullup *et al.* 2013; Wang *et al.* 2016). Thus, uncovering mechanisms that suppress the autophagy defect, provides insights into the pathogenesis and also therapeutic treatments of Vici syndrome.

## Systemic regulation of autophagy

In multicellular organisms, autophagy activity is systemically coordinated to ameliorate deleterious effects elicited by locally imposed stresses such as nutrient restriction, and also to maintain cellular homeostasis at an organismal level (Fenouille *et al.* 2017). In *C. elegans*, a circuit has been revealed that senses and transduces cuticle damage to elicit a long-range organism-wide autophagy response (Zhang *et al.* 2019). The outer layer of the cuticle in *C. elegans* contains two discrete interacting groups of collagens that constitute alternating parallel circumferential bands, known as annuli and annular furrows (McMahon *et al.* 2003). Loss of function of annular furrow collagen genes activates autophagy in multiple tissues, including the hypodermis, intestine, and muscle (Zhang *et al.* 2019). This systemic response is sensed and triggered by sensory neurons with cuticle-embedded cilial endings. The TGF-β-like molecule DAF-7/TGF-β, secreted from the ASI pair of ciliated neurons, activates a canonical TGFβ signaling pathway in distant tissues to induce autophagy (Zhang *et al.* 2019). Amino acids modulate the activity of the metabotropic glutamate receptor homologs MGL-1 and MGL-2 in the AIY and AIB interneurons, respectively, to regulate systemic autophagy responses (Kang and Avery 2009), but the circuit mediating this response has yet to be determined.

## Selective autophagy

Protein aggregates or damaged/superfluous organelles can be selectively removed by autophagy (Stolz *et al.* 2014). A family of receptor proteins that simultaneously bind to cargo and Atg8/LC3 is essential for recognizing specific cargos for degradation. For example, SQST-1/p62, which contains a self-oligomerization PB1 domain, an LC3-interacting LIR motif and a UBA ubiquitin binding domain, functions as a receptor to mediate aggregation, and autophagic degradation of ubiquitinated misfolded proteins (Stolz *et al.* 2014; Gatica *et al.* 2018; Lamark and Johansen 2021). Studies of autophagic degradation of protein aggregates and paternal mitochondria during *C. elegans* embryogenesis have impacted our understanding of selective autophagy.

## Degradation of P granule components in somatic cells

### Receptor and scaffold protein are involved in degradation of PGL granules

During early embryogenesis, the oocyte-derived P granules, which are specialized protein-RNA aggregates, become localized exclusively in the germ blastomeres P1, P2, P3, and P4 during asymmetric cell divisions, and eventually in the two germ precursor cells, Z2 and Z3, derived from P4 (Strome 2005). One mechanism for such asymmetric localization is rapid disassembly and removal of P granules that are partitioned into somatic blastomeres (Hird *et al.* 1996; DeRenzo *et al.* 2003). The RGG box-containing P granule proteins PGL-1 and PGL-3 (collectively called PGL proteins) are degraded by autophagy in somatic cells, but accumulate into a large number of aggregates, called PGL granules, in autophagy mutants (Fig. 4, a and b) (Zhang *et al.* 2009). Genetic screens have identified that *sepa-1* and *epg-2* are required for removal of PGL proteins (Zhang *et al.* 2009; Tian *et al.* 2010a) (Fig. 5). In *sepa-1* mutant embryos, PGL proteins fail to be removed and are diffusely localized in the cytoplasm of somatic cells (Zhang *et al.* 2009), while in *epg-2* mutants, PGL proteins form aggregates but are not colocalized with LGG-1-positive autophagic structures (Tian *et al.* 2010a). SEPA-1 self-oligomerizes and also interacts with PGL-3 and LGG-1, acting as the receptor for formation and degradation of PGL granules (Zhang *et al.* 2009). EPG-2 also self-oligomerizes and functions as a scaffold by directly interacting with SEPA-1 and multiple Atg proteins (Tian *et al.* 2010a). SEPA-1 and EPG-2 are zygotically synthesized and display characteristic temporal expression patterns. SEPA-1 aggregates are detectable at the ∼24-cell stage, peak at the ∼100 cell stage and become undetectable at the comma stage. The temporal EPG-2 expression pattern partially overlaps with SEPA-1 (Noda *et al.* 2020). SEPA-1 and EPG-2 are degraded by autophagy in a manner independent of PGL granules (Zhang *et al.* 2017b).

### Phase separation and transition control assembly and degradation of PGL granules

Autophagy activity occurs at a basal level during embryogenesis and presumably the autophagosomes are relatively uniformly sized. Removal of the diffuse oocyte-loaded PGL-1/-3 proteins requires mechanisms that ensure the coordination of the assembly rate and size of PGL granules with autophagic flux during embryogenesis (Wang and Zhang 2019). The assembly of protein aggregates can be driven by LLPS. The resultant liquid-like aggregates further undergo phase transition to form gel-like or solid structures (Wang and Zhang 2019; Noda *et al.* 2020). SEPA-1 and EPG-2 act concertedly to specify phase separation and transition of PGL proteins (Zhang *et al.* 2018). In vitro LLPS assays showed that PGL-1/-3/SEPA-1 undergo LLPS and the resultant droplets exhibit liquid-like properties, including fusion capability and highly mobile interior molecules (Zhang *et al.* 2018). SEPA-1 acts in a concentration-dependent manner to lower the critical concentration of PGL-1/-3 for LLPS. SEPA-1 homogenously disperses into PGL-1/-3 droplets (Zhang *et al.* 2018). PGL-1/-3 levels are gradually reduced as development proceeds during embryogenesis (Zhang *et al.* 2009). The increased SEPA-1 level promotes condensation of low levels of diffuse PGL-1/-3 into aggregates for degradation. Addition of EPG-2 leads to transition of PGL-1/-3/SEPA-1 droplets to a gel-like state, which show low mobility and remain smaller in size over time. EPG-2 coats the surface of PGL granules (Zhang *et al.* 2018). In autophagy mutants, EPG-2 encircles PGL granules and reduces the mobility of PGL proteins (Zhang *et al.* 2018). Missense mutations in PGL-1 that promote gelation of PGL granules render their degradation independent of EPG-2 (Zhang *et al.* 2018). Thus, the gel state of PGL granules is essential for their autophagic degradation.

### Stress-controlled RNA recruitment switches the fate of PGL granules from autophagic degradation to accumulation

Surprisingly, in embryos laid by animals grown under heat-stress conditions, PGL proteins escape autophagic degradation and accumulate into a large number of granules to confer a stress adaptation function (Zhang *et al.* 2018). mTORC1, but not mTORC2, is required for PGL granules to evade degradation (Zhang *et al.* 2018). LET-363/mTOR phosphorylates PGL-1/-3, whose level is significantly elevated in embryos laid under heat-stress conditions. Phosphorylated PGL-1/-3 facilitate LLPS. EPG-2 still undergoes autophagic degradation under heat-stress conditions. Its level does not correlate with the enhanced rate of PGL granule formation and is not sufficient to make PGL granules amenable to degradation (Zhang *et al.* 2018). The insufficient targeting of EPG-2 appears to be due to partitioning of mRNAs into PGL granules (Zheng *et al.* 2023). In autophagy mutants, mRNAs are absent from PGL granules formed at normal temperatures. PGL granules formed under heat-stress conditions are enriched in mRNAs (Zheng *et al.* 2023). In the in vitro LLPS assay, mRNAs promote the formation and liquidity of PGL granules, and also inhibit the recruitment of EPG-2. Depleting factors involved in mRNA processing, transport or translation reduces the recruitment of mRNAs, resulting in the recruitment of EPG-2 to PGL granules and subsequent autophagic degradation (Zheng *et al.* 2023). Therefore, sorting of RNAs into PGL granules controls the recruitment of EPG-2, acting as a switch for their accumulation to provide a fitness advantage under heat stress.

## SQST-1/p62 and miRISC

The *C. elegans* SQSTM1/p62 homolog SQST-1 is removed by autophagy. SQST-1/p62 is diffusely distributed at a low level in the cytoplasm in wild-type embryos, but accumulates into a large number of aggregates in autophagy mutants (Tian *et al.* 2010a). The self-oligomerizing protein EPG-7 functions as the scaffold protein for degradation of SQST-1/p62 (Lin *et al.* 2013). EPG-7 directly interacts with SQST-1/p62 and also associates with multiple autophagy proteins, including LGG-1, LGG-3/Atg12, ATG-18, and ATG-9 (Lin *et al.* 2013). The scaffold protein may recruit core Atg proteins to trigger the formation of surrounding autophagosomal membranes.

The miRNA-induced silencing complex (miRISC) in *C. elegans* contains Argonaute (Ago), miRNA, and AIN-1, a member of the GW182 family of proteins (Zhang and Zhang 2013). Compared to wild-type animals, AIN-1 is present at higher levels in autophagy mutants and accumulates into a large number of aggregates that colocalize with SQST-1/p62 aggregates. Degradation of AIN-1 also requires EPG-7 (Zhang and Zhang 2013). Therefore, autophagy participates in diverse miRNA-regulated biological processes by controlling the miRISC level.

## Clearance of paternal mitochondria and MOs

Animal offspring only inherit the mitochondrial genome (mtDNA) from the female parent (Al Rawi *et al.* 2011; Sato and Sato 2011). In *C. elegans*, paternal mitochondria and mtRNA are selectively degraded by autophagy (Al Rawi *et al.* 2011; Sato and Sato 2011). In two- and four-cell-stage embryos, paternal mitochondria are partitioned into blastomeres, then they are gradually eliminated, becoming almost undetectable at the 16-cell stage. In autophagy mutants, they persist in late-stage embryos and L1 larvae (Sato and Sato 2011). The specialized sperm-specific post-Golgi organelles, called membranous organelles (MOs), are also degraded by autophagy (Al Rawi *et al.* 2011). Degradation of paternal mitochondria and MOs (collectively called paternal organelles) is known as allophagy [i.e. allogeneic (nonself) organelle autophagy] (Sato *et al.* 2018). After fertilization, paternal mitochondria and MOs triggers the recruitment of the receptor ALLO-1 for autophagic degradation. Loss of function of *allo-1* causes accumulation of paternal organelles (Sato *et al.* 2018). ALLO-1 has two isoforms (i.e. ALLO-1a and ALLO-1b) with different C-terminal sequences. MOs and paternal mitochondria are recognized by ALLO-1a and ALLO-1b, respectively (Sasaki *et al.* 2024). Successful allophagy depends on IKKE-1, a *C. elegans* homolog of the TBK1 and IKKε kinases. IKKE-1 interacts and phosphorylates ALLO-1 and/or autophagy-related proteins, for example, EPG-7/ATG-11, resulting in accumulation of ALLO-1 and autophagy-related proteins (e.g. EPG-7) around the cargo (Sato *et al.* 2018; Sasaki *et al.* 2024). ALLO-1 and IKKE-1 are maternally expressed (Sato *et al.* 2018). After fertilization, MOs are rapidly ubiquitinated, while the ubiquitination status of paternal mitochondria is unclear (Al Rawi *et al.* 2011; Sato *et al.* 2018; Molina *et al.* 2019). Paternal mitochondria selectively express the ubiquitin-independent mitophagy receptor-FNDC-1 (*C. elegans* ortholog of FUNDC1), whose loss of function specifically delays the elimination of paternal mitochondria, but not MOs (Lim *et al.* 2019). The inner mitochondrial membrane protein PHB2 acts as a receptor for autophagic degradation of damaged mitochondria. Paternal inactivation of *phb-2* in *C. elegans* causes accumulation of paternal mitochondria (Wei *et al.* 2017). Thus, multiple mechanisms utilizing different receptors are involved in autophagic degradation of paternal mitochondria in fertilized embryos.

## Monitoring autophagy in *C. elegans*

Multiple methods, including analyzing the dynamics of LGG-1 and LGG-2 reporters (e.g. GFP::LGG-1, GFP::LGG-2), western blot assay and immunofluorescence analysis of LGG-1 and LGG-2, analyzing the degradation of autophagy substrates (e.g. SQST-1/p62, PGL granules), transmission electron microscopy (TEM), and Correlative Light and Electron Microscopy have been developed to monitor autophagy in *C. elegans*. These methods have been extensively described (Jenzer *et al.* 2015; Zhang *et al.* 2015a; Chen *et al.* 2017b; Largeau and Legouis 2019; Springhorn and Hoppe 2019). Here, we only give a brief introduction about the widely used methods to monitor autophagy in *C. elegans*.

## The LGG-1 reporter

### GFP::LGG-1

The Atg8/LC3 reporter is widely used to monitor autophagy in yeast, *C. elegans* and mammalian cells (Meléndez *et al.* 2003; Klionsky *et al.* 2021). GFP::LGG-1 forms puncta in various cell types during development. The GFP signal is quenched in acidic environments. Thus, GFP::LGG-1-labeled puncta represent IMs, autophagosomes, and unacidified amphisomes (Zhang *et al.* 2015a; Chang *et al.* 2017). The dual-fluorescent mCherry::GFP::LGG-1 reporter labels these structures (detected as mCherry/GFP double-positive puncta), and also acidified amphisomes and autolysosomes (detected as mCherry-only puncta) (Chang *et al.* 2017). The number of LGG-1-positive puncta can be increased by induction of autophagy or by a block in autophagy downstream of autophagosome initiation (Zhang *et al.* 2015a; Chang *et al.* 2017). The LGG-1 reporter can also be incorporated into protein aggregates (Zhang *et al.* 2015a). Additional methods described below should also be performed to confirm the occurrence of autophagy.

### Degradation of autophagy substrates

The removal of autophagy substrates including PGL granules, SQST-1 aggregates, and paternal organelles can be used to evaluate autophagy activity in embryos (Zhang *et al.* 2015a). Accumulation of SQST-1/p62 aggregates at larval and adult stages may be due to elevated expression of SQST-1/p62 or targeting of SQST-1/p62 to aggregates formed by misfolded proteins or damaged organelles (Zhang *et al.* 2015a). Thus, caution is needed when autophagic flux is monitored in larval and adult animals. Additional assays, for example, quantifying the number of GFP::LGG-1 puncta, should be used to examine autophagy (Zhang *et al.* 2015a; Chang *et al.* 2020).

### Transmission electron microscopy

The ultrastructure of autophagic membranes/vesicles and also the engulfed cytoplasmic contents can be directly visualized by TEM (Meléndez *et al.* 2003, 2008; Tian *et al.* 2010a; Wu *et al.* 2015; Zhang *et al.* 2015a). TEM analysis revealed that different stages of autophagic structures accumulate in different autophagy gene mutants. For example, IMs, which are extremely rare in wild-type embryos, accumulate in *epg-3*, *epg-4*, *epg-6*, *atg-2*, and *atg-18* mutant embryos (Tian *et al.* 2010a; Lu *et al.* 2011). Small autophagosomes are formed in *lgg-2* mutant embryos, while *epg-5* mutants accumulate unfused autophagosomes and nondegradative autolysosomes (Wu *et al.* 2015; Wang and Zhang 2019). The unambiguously identification of specific autophagic structures confirmed the molecular function of these autophagy genes in the pathway.

## Autophagy in stress

Autophagy occurs at basal levels in nearly all cell types, but it is increased by diverse intracellular and extracellular cues or in the adaptation to environmental stress. All tissues carry out autophagy (Chapin *et al*. 2015; Chang *et al.* 2017). The first documentation of a role of autophagy in a multicellular organism in response to stress reported that autophagy genes were required for animals to undergo the remodeling that occurs during dauer formation (Meléndez *et al.* 2003) (see Autophagy in development section). Under high temperature, high population density or starvation conditions, young larvae (past the L1 stage) arrest as dauers, a specialized state in which animals can survive for several months (Cassada and Russell 1975; Albert *et al.* 1981; Golden and Riddle 1982). Dauer animals change their physiology and metabolism to survive long term. Autophagy is also required for the survival of newly hatched L1 larvae upon starvation (Kang *et al.* 2007).

Nearly, two decades of research involving the genetic ablation of the autophagy machinery in diverse animal models or cell culture systems has shown how autophagy is a central mechanism by which cells response to stress stimuli, such as starvation, metabolic stress, heat stress, oxidative stress, hypoxia, and exercise (Levine and Kroemer 2019; Mizushima and Komatsu 2011).

In *C. elegans*, a mild heat shock (1 h at 36°C) when applied to young adult animals triggers a hermetic response, triggers autophagy, and extends lifespan (Kumsta *et al.* 2017). A severe and more acute heat shock (1 h at 37°C) of young adults, or L4 larvae, induces the transient fragmentation of mitochondria, the formation of aggregates in the matrix and mitophagy, a DCT-1-, PINK-1-, and PDR-1-dependent process (Palikaras *et al.* 2015; Chen *et al.* 2021b). In response to acute stress, autophagosomes form on mitochondria and protect larvae from acute heat shock and facilitate the mitochondrial rebuilding via a DRP-1-dependent process (Chen *et al.* 2021b), suggesting a functional link between mitochondrial fission and autophagosomal biogenesis. The adaptation to acute heat shock does not appear to require PDR-1, PINK-1, DCT-1, or FNDC-1 (FUN14 domain containing 1, proteins involved in various selective mitophagy processes) (Chen *et al.* 2021b). Thus, part of the fragmented mitochondria can be resulting from a yet unidentified mitophagy pathway or through bulk autophagy. Studies in *C. elegans* muscle cells exposed to various stresses suggest a balance response between PINK-1/PDR-1-dependent mitophagy and mitochondrial biogenesis (Palikaras *et al.* 2015) (see mitophagy in Longevity section).

Hypoxia in *C. elegans* results in an increase in LGG-1-positive fluorescently labeled foci (Samokhvalov *et al.* 2008). RNAi depletion of *bec-1/Becn1*, *lgg-1,lgg-2,* or a mutation in *unc-51/Atg1* decreased animal survival after severe hypoxic injury (Samokhvalov *et al.* 2008). The hypersensitivity phenotype of *bec-1/Becn1* RNAi-depleted animals could be blocked by loss-of-function mutations in either the apoptosis (*ced-3*) or necrosis (*crt-1*) pathway (Samokhvalov *et al.* 2008). Thus, it appears that inhibition of autophagy sensitizes animals to hypoxic injury and that this sensitization is no longer functional when either of the two cell death mechanisms are inhibited (Samokhvalov *et al.* 2008).

A small increase of glucose in the *C. elegans* diet results in a shortened lifespan (Lee *et al.* 2009). Excess glucose levels increase the levels of glycerol-3-phosphate (Gro3P), a key metabolite in lipid and carbohydrate metabolism. The accumulation of Gro3P, following excess nutrients can cause metabolic stress, and increase the production of reactive oxygen species (ROS) to damage macromolecules, and cellular dysfunction (Prentki and Madiraju 2008; Prentki *et al.* 2020). The glycerol-3-phosphate phosphatase (G3PP) operates a glycerol shunt as it hydrolyzes glucose derived GroP to glycerol (Possik *et al.* 2022). In *C. elegans*, there are three homologs of the G3PP enzymes (PGPH-1,2,3) and they act in glycerol synthesis and protection from stress (Possik *et al.* 2022). Activation of PGPH-2, the major G3PP worm homolog, mimics some of the beneficial effects of caloric restriction (CR), particularly under glucotoxic conditions (Possik *et al.* 2023). In *C. elegans*, overexpression of PGPH-2/G3PP, under glucose excess, depletes glycogen stores to activate AMP-activated protein kinase (AMPK), which leads to the nuclear translocation of HLH-30/TFEB, the induction of autophagy and increases lifespan under glucotoxicity (Possik *et al.* 2023). *pgph-2* overexpression extends lifespan and healthspan independently of *daf-16* and the insulin-like signaling pathway (Possik *et al.* 2023). Autophagy is induced in the intestine of *pgph-2* overexpressing animals, but it is not clear if there is any selective autophagy involved. Thus, stress stimuli can elicit different responses that cooperate to promote optimal cellular repair and adaptation to stress. A diverse range of stressors modulate autophagy at different levels, transcriptional, posttranslational or at the cellular level. To understand whether general bulk autophagy or selective forms of autophagy are involved in every condition still will require future studies.

## Autophagy in development

The first role for autophagy in the development of a multicellular organism was documented in dauer development (Meléndez *et al.* 2003). When young larvae are faced with a lack of nutrients, high temperature, or over-crowded conditions, animals arrest as dauers. However, insulin IGF-1/*daf-2* signaling mutants when grown at the restrictive temperature form constitutive dauer. Employing *daf-2/IIR* or *daf-7/TGF-β* mutants and exposing them to the restrictive temperature where they would become dauers, was used to show that autophagy genes were required for the remodeling that occurs during dauer development (Meléndez *et al.* 2003). In this first screen for mutants that were required for dauer formation, RNAi depletion (by injection) of *daf-2/IIR* mutants showed that *unc-51/ULK*, *bec-1/Becn1*, *atg-7/Atg7*, *lgg-1/Atg8*, and *atg-18/Wipi2* were required for dauer larvae formation (Meléndez *et al.* 2003). This was the first demonstration of a role for autophagy in the remodeling that is associated with dauer formation. During dauer development, GFP::LGG-1 expression was found to change from a diffused subcellular localization to increased GFP::LGG-1 positive punctate or foci (Meléndez *et al.* 2003). Similarly, GFP::LGG-2 presents a diffused localized expression that forms GFP::LGG-2 positive punctate foci during dauer development (Alberti *et al.* 2010). In a set of experiments to investigate in what tissues *atg-18* is required for the increase in lipids that is associated with the dauer phenotype of *daf-2/IIR* mutants, transgenic expression of *atg-18* in neurons rescued the phenotype of *daf-2; atg-18* mutants (Jia *et al.* 2019). However, this experiment only examined the tissue specificity of *atg-18* in fat metabolism of *daf-2/IIR* mutant dauer larvae. The tissues that require *atg-18* in wild-type animals and in other developmental stages remain not known. Whether autophagy genes are required in all tissues or if they act non cell autonomously to promote the remodeling that occurs in dauer animals is not clear. It is also not known if autophagy gene activity is required for the behavior associated with dauer formation, such as nictation.

## Autophagy in neurons

Neurons, like all cells, rely on autophagy to efficiently remove toxic materials, damaged organelles, and cellular debris. Several reviews of the role of autophagy specifically in neurons have recently been published (Stavoe and Holzbaur 2019; Hill and Colon-Ramos 2020; Fleming *et al.* 2022; Sidibe *et al.* 2022).

## Compartmentalization of neurons

Neurons are polarized and postmitotic cells, and as such have no ability to dilute damaged constituents, such as damaged organelles or accumulated proteins, through cell division. Because of their function in the processing and transmission of information, neurons have high metabolic demands that, in some cases, are far removed from the cell body, in the axons, or dendrites. Thus, autophagy is particularly significant for neurons in the removal of aggregated or damaged proteins and organelles. Studies with fluorescent markers have established autophagosome biogenesis at the axonal tip and retrograde transport of autophagosomes to the cell body (Lee *et al.* 2011; Maday *et al.* 2012; Maday and Holzbaur 2014; Cheng *et al.* 2015). Interestingly, autophagosome formation in axons is independent from input from the cell body and can occur in severed axons (Hernandez *et al.* 2012; Soukup *et al.* 2016). Like in most cells, autophagy is induced under stress conditions but appears to be constitutively active at basal levels (Xilouri and Stefanis 2010; Lee 2012; Wong and Holzbaur 2015). These basal levels seem to be required for neuronal survival because the disruption of autophagic flux results in axon degeneration and neuronal cell death in mammals (Hara *et al.* 2006; Komatsu *et al.* 2007; Yue *et al.* 2009; Yang *et al.* 2013b).

**Fig. 6.**
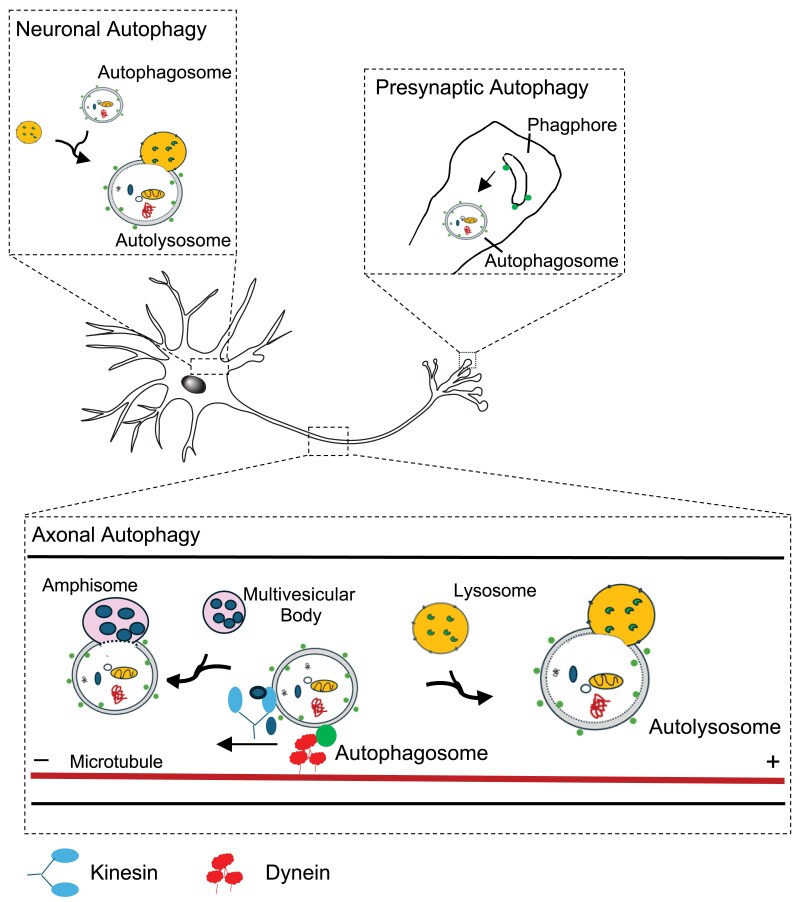
Autophagy in Neurons. Autophagosome biogenesis is compartmentalized in neurons. Autophagosomes are formed at axonal terminals near presynaptic compartments. Retrograde transport of autophagosomes occurs toward the cell body and this process is linked to the autophagosome acidification and maturation. Acidification requires fusion with proteolytic lysosomes or the fusion of endosomes (multivesicular bodies) with autophagosomes to form amphisomes. Autophagosomes rely on microtubules and associate with the minus-end-directed motors like dynein (in red) to move toward the cell body. This figure is inspired by figures from (Hill and Colon-Ramos 2020). For a more detailed description of proteins involved in the transport of autophagosomes, see Stavoe and Holzbaur (2019) and Hill and Colon-Ramos (2020)).

Autophagy is important for many aspects of neuronal physiology, including neurotransmitter receptor turnover (Rowland *et al.* 2006), synaptic development (Shen and Ganetzky 2009; Stavoe *et al.* 2016), structural, and functional synaptic plasticity, which in turn is required for learning and memory (Nikoletopoulou *et al.* 2017; Glatigny *et al.* 2019), and synaptic pruning (Tang *et al.* 2014). Autophagosome biogenesis has been observed at synaptic sites in the distal axons of the interneuron AIY, which receive and process synaptic input from amphid sensory neurons and modulate behavioral plasticity as they respond to different sensory modalities, including gustatory, olfactory, and thermal information (Ishihara *et al.* 2002; de Bono and Maricq 2005; Chalasani *et al.* 2007; Satoh *et al.* 2014). In a screen for mutants that disrupted presynaptic development of AIY interneurons, an *atg-9* mutation was recovered (Stavoe *et al.* 2016). Moreover, autophagosome biogenesis is spatially organized such that autophagosomes form in distal axonal compartments near the synapses (Fig. 6) (Stavoe *et al.* 2016). The autophagosomes undergo retrograde transport and fusion with late endosomes and lysosomes before degradation occurs in the cell body (Stavoe *et al.* 2016; Hill *et al.* 2019).

The fusion of autophagosomes with late endosomes and lysosomes appears to increase acidification, thus autophagosomes in the axons lack degradative capacity, until they have been transported to the soma (Fig. 6). Blocking the retrograde transport of autophagosomes blocks acidification and their capacity to degrade cargo (Fu *et al.* 2014; Wong and Holzbaur 2014). Mutants in several autophagy genes displayed defects in active zone assembly and synaptic vesicle clustering in early larval stages (Stavoe *et al.* 2016; Stavoe and Holzbaur 2019). Mutants in genes involved in selective forms of autophagy did not phenocopy mutants required for bulk autophagy mutants, suggesting that bulk autophagy rather than selective autophagy is required in axons (Stavoe *et al.* 2016; Stavoe and Holzbaur 2019). In addition, autophagy did not appear to be required for general neuronal development, although autophagy was found to control the rate of axon outgrowth but not dendritic patterning of the highly elaborate dendritic arbors of PVD somatosensory neurons (Stavoe *et al.* 2016; Stavoe and Holzbaur 2019). Thus, autophagy may regulate different stages of development in specific neurons, rather than serving a general function in all neurons (Stavoe *et al.* 2016; Stavoe and Holzbaur 2019). It should be noted that neurons in *C. elegans* are nonmyelinated and that autophagy may still function in mammalian myelinated neurons.

## Presynaptic autophagy

The ATG-9 protein is transported from the neuronal cell body to presynaptic sites via the synaptic vesicle kinesin, UNC-104/KIF1A (Fig. 6) (Stavoe *et al.* 2016). In vivo and electron microscopic studies showed that ATG-9 localized to a subpopulation of vesicles at synapses, and colocalized with RAB-3 and SNG-1/synaptogyrin in vesicles that emerge from the Golgi apparatus via the AP-3 complex. Synaptic vesicle endocytosis of ATG-9 required *unc-26/synaptojanin 1*, *unc-57/endophilin A*, and *dyn-1/dynamin* activity (Stavoe *et al.* 2016). At presynaptic sites, ATG-9 rich vesicles undergo endo-exocytosis using the synaptic vesicle cycling machinery (Yang *et al.* 2022). This exocytosis is dependent on the endocytic genes *unc-26*/synaptojanin 1, *unc-57*/endophillin A and *dyn-1*/dynamin, since abnormal ATG-9 accumulation was observed in subsynaptic clathrin-rich foci (Yang *et al.* 2022). A mutation in *unc-26/synaptojanin 1*, a phosphatase that is associated with early onset of Parkinson's disease in humans, resulted in the accumulation of ATG-9 in presynaptic nerve terminals, defects in neurotransmission and locomotion. Similarly, loss-of-function alleles in autophagy genes that act in the early steps of autophagosome formation also affected the localization of ATG-9, resulting in its accumulation at presynaptic sites. Thus, ATG-9 exo-endocytosis mechanistically links the biogenesis of autophagosomes and the activity-dependent synaptic vesicle cycle (Yang *et al.* 2022). In a screen for mutations that disrupt ATG-9 sorting at synapses, the long isoform of the active zone protein Clarinet (CLA-1L) was found to be necessary for presynaptic sorting of ATG-9 and activity-dependent autophagosome formation (Xuan *et al.* 2023). Specifically, the ATG-9 protein, but not synaptic vesicle proteins, abnormally accumulate to subsynaptic regions enriched for clathrin in *cla-1(L)* mutants (Xuan *et al.* 2023). This mislocalization of ATG-9 is suppressed by mutants for synaptic vesicle exocytosis, suggesting that the ATG-9 phenotype in *cla-1(L)* mutants results from defects in ATG-9 sorting during exo-endocytosis. However, only activity-induced autophagy, and not basal autophagy is affected in *cla-1(L)* mutants, suggesting that other molecules are redundant with CLA-1(L) in supporting basal levels of autophagy. The clathrin adaptor complexes, AP-1, AP-2, and AP180, regulate ATG-9 sorting at presynaptic sites (Xuan *et al.* 2023), and via SDPN-1/syndapin-dependent vesicles. These results are consistent with findings in nonneuronal cells, where AP-1 and AP-2 complexes are required to traffic ATG-9 between the plasma membrane, the trans-Golgi network, recycling endosomes and the forming autophagosome (Guo *et al.* 2012; Puri *et al.* 2014; Imai *et al.* 2016; Zhou *et al.* 2017). Interestingly, the sorting of ATG-9 at synapses is genetically separable from the sorting of synaptic vesicle proteins (Xuan *et al.* 2023). Both, ATG-9 and synaptic vesicles require the activity of the synaptic vesicle kinesin UNC-104/KIF-1A. A model has been proposed whereby CLA-1L bridges the exocytic active zone regions with the endocytic periactive zones to regulate presynaptic sorting of ATG-9 (Xuan *et al.* 2023).

In *C. elegans*, autophagy may selectively regulate the surface expression of GABA_A_ receptors because (1) GABA_A_ receptors colocalized with autophagosome markers, and (2) autophagy was found to reduce GABA_A_ receptor surface expression in noninnervated muscle (Rowland *et al.* 2006). Autophagy may modulate neuronal excitation (Rowland *et al.* 2006). In neurons, the autophagy machinery is tightly coupled to neuronal activity, and autophagosome biogenesis occurs near presynaptic sites and in response to increased neuronal activity (Bunge 1973; Katsumata *et al.* 2010; Maday *et al.* 2012; Shehata *et al.* 2012; Soukup *et al.* 2016; Stavoe *et al.* 2016; Vijayan and Verstreken 2017; Hill and Colon-Ramos 2019; Kulkarni *et al.* 2021; Soykan *et al.* 2021).

### Autophagy in axon growth

**Fig. 7.**
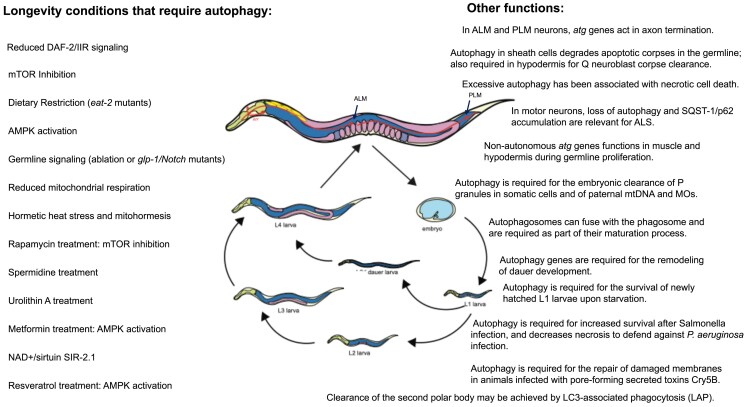
The role of autophagy in longevity and cellular homeostasis. On the left, longevity pathways that include the Insulin IGF-1 Receptor signaling (IIS), inhibition of TOR, dietary restriction, germline-less mutants, and reduced mitochondrial respiration. All have been shown to induce autophagy and require autophagy for their improvement on healthspan and longevity. Although the mechanisms are not well understood, several positive regulators are known to induce autophagy: the forkhead transcription factors DAF-16/FOXO and PHA-4/FOXA, the energy sensor AMPK, the histone deacetylase SIRT-1, the HLH-30/TFEB, and the heat shock factor HSF-1 (see Table 2). Listed are also several pro-longevity treatments that have been found to require autophagy. On the right, examples of the role of autophagy genes or the autophagy process in other cellular and developmental programs, as discussed in this review.

RPM-1 is an atypical RING E3 ubiquitin ligase in the PAM/Highwire/RPM-1 (PHR) protein family and functions to degrade UNC-51/ULK and generally restricts autophagy in the nervous system (Crawley *et al.* 2019). RPM-1 localizes to the growth cone of mechanosensory neurons, where it regulates axon termination (Schaefer *et al*. 2000; Grill *et al.* 2007; Borgen *et al.* 2017). For example, in *rpm-1* mutants, the ALM and PLM mechanosensory neurons fail to terminate axon growth (Fig. 7) (Schaefer *et al*. 2000). In contrast, *unc-51* mutants displayed a premature axon termination phenotype, and *rpm-1; unc-51* double mutants showed complete suppression of the failed termination phenotype, indicating that RPM-1 functions upstream of UNC-51 (Crawley *et al*. 2019). Loss of *rpm-1* activity resulted in the stabilization of UNC-51/ULK protein levels, indicating that RPM-1 spatially regulates the stability of UNC-51/ULK in axons, and broadly across the nervous system (Crawley *et al*. 2019). Therefore, RPM-1 may inhibit autophagosome formation in specific axonal compartments in *C. elegans*. In vertebrates, two other ubiquitin ligases, TRAF6 and KLHL20, regulate ULK1 also in nonneuronal cells, but it is not known whether they or RPM-1/Highwire act to regulate ULK in the vertebrate nervous system (Nazio *et al*. 2013). Finally, RPM-1 directly affected autophagy levels, as the defects in axon termination and synapse maintenance in *rpm-1* loss-of-function mutants were dependent on multiple autophagy genes, including the *bec-1/Becn1*, *epg-8/Atg14, atg-9*, and *epg-6/Wipi* (Crawley *et al*. 2019). Work in cell culture and in mammalian neurons has also shown that extension of neuronal processes is regulated by autophagy, suggesting that these mechanisms are conserved (Ban *et al*. 2013; Chen *et al*. 2013).

### Exophers in neurons

In *C. elegans* adult neurons inhibiting chaperone protein expression, proteasome activity, or the disruption of autophagy function, can trigger the production of large membrane-surrounded vesicles, which contain aggregates and organelles and are referred to as exophers (Melentijevic *et al*. 2017; Nicolas-Avila *et al*. 2022). Exophers were first discovered in *C. elegans* and have been characterized as giant extracellular vesicles, which may be as large as the neuronal somata from which they are extruded from (Melentijevic *et al*. 2017). Exopher production is increased by various cellular stressors, such as proteosome inhibition, disruption of autophagy, increased oxidation, increased osmotic strength, nutrient deprivation, expression of aggregating Huntingtin polyQ protein (HTT-Q128), or mCherry proteins (Melentijevic *et al*. 2017; Cooper *et al*. 2021). Exophers selectively remove aggregated proteins and have been reported in mammalian systems (Davis *et al*. 2018; Choong *et al*. 2021; Nicolas-Avila *et al*. 2021, 2022). In *C. elegans*, it was noted that neurons that produced exophers under HTT-Q128 protein expression were better functioning, suggesting that exopher production serves a protective function (Melentijevic *et al*. 2017).

Exophers can be produced by the mechanosensory touch receptor neurons, and of the six touch neurons, the highest frequency of exophers is produced by the ALMR neuron (Melentijevic *et al*. 2017; Arnold *et al*. 2020; Cooper *et al*. 2021). Once exophers are released, they are engulfed by the surrounding hypodermis (Hyp7), and are broken up into smaller vesicles that are referred to as a “starry night” process (Melentijevic *et al*. 2017; Arnold *et al*. 2020, 2023; Wang *et al*. 2023). Dysruption of autophagy, proteosome activity, or chaperone protein expression, enhanced exopher production. A recent report found that the autophagy protein ATG-16.2 mediates the beneficial effects of inhibition of early-acting autophagy genes in neurons (Yang *et al*. 2024). They found that knockdown of early-acting autophagy genes increased exopher production, decreased neuronal PolyQ aggregates, and extended lifespan, independently of autophagic degradation. An exception of the early acting genes was *atg-16.2*, as *atg-16.2* null animals did not show the exopher production increase or the phenotypes associated with knockdown of the early acting autophagy genes. Rescue experiments showed that the WD40 domain of ATG-16.2 mediates the beneficial effects of inhibiting early-acting autophagy genes in *C. elegans* neurons (Yang *et al*. 2024).

Vesicles in the hypodermis acquire phagosome maturation markers (2XFYVE/Hrs, a biosensor for PI3P, recycling early phagosome and endosomes RAB-10, and late phagosome and endosome RAB-7), before they are eventually degraded by hypodermal lysosomes (Wang *et al*. 2023). The hypodermis acts as an exopher phagocyte and requires hypodermal actin, Arp2/3, and the phagosome maturation factors SAND-1/Mon1, GTPase RAB-35, CNT-1 ARF-GAP, and the microtubule motor-associated GTPase ARL-8 (Wang *et al*. 2023). In addition, the GTPase ARF-6 and its effector SEC-10/exocyst activity were found to be required in the hypodermis, together with the CED-1 phagocytic receptor to generate exophers (Wang *et al*. 2023). A GFP::LGG-1 reporter colocalized with the “starry night” phagosomes, and degradation required CUP-5 activity, indicating that lysosome fusion is a late step in the process (Wang *et al*. 2023), even if lysosomal activity was not obviously required for exopher-phagosome resolution into smaller vesicles. Since the “starry night” vesicles acquired LGG-1/LC3, after fragmentation, the exopher phagosome fragments are thought to fuse with autophagosomes. Alternatively, exopher degradation requires a form of LC3-associated phagocytosis (LAP), similar to the case of neuronal pruning by phagocytic glia in vertebrates (Kim *et al*. 2017; Lieberman *et al*. 2019; Meng *et al*. 2022).

### Autophagy in a model for amyotropic lateral sclerosis

Amyotropic lateral sclerosis (ALS) is an incurable neurodegenerative disease characterized by the adult-onset of progressive neuromuscular denervation and motor neuron loss. Mutations in the fused in sarcoma (fus) gene account for 5% familial and 1% sporadic ALS cases (Lagier-Tourenne *et al*. 2010; Lagier-Tourenne and Cleveland 2010). Cytoplasmic FUS misexpression may also cause sporadic ALS (Tyzack *et al*. 2019) and cytoplasmic FUS aggregates have been observed in patients with frontotemporal dementia. To establish a model for ALS in *C. elegans*, mutations equivalent to the human disease-causing missense mutations in ALS patients (*fust-1R524S^C^* and *fust-1P525L^C^*) were generated in the *C. elegans* FUS gene *fust-1* (Baskoylu *et al*. 2022). Mutant animals showed hypersensitivity to stress and impaired neuromuscular function (Baskoylu *et al*. 2022). Specifically, these animals displayed FUST-1 accumulation, defects in basal autophagy levels and SQST-1/p62 accumulation in motor neurons. Loss of the autophagy adaptor protein *sqst-1/p62,* suppressed the stress-induced locomotion and aldicarb sensitivity of the ALS/FUS mutant animals, but did not suppress the defects in basal autophagy defects (Baskoylu *et al*. 2022), suggesting that the autophagy defect, which leads to SQST-1/p62 accumulation is relevant for the severity of ALS. These findings suggest that stress, in addition to defective autophagy in ALS, *fust-1* mutants, result in SQST-1/p62 accumulation to drive neuromuscular junction defects. How SQST-1/p62 accumulation perturbs neuronal function is not known, but these results suggest that the autophagy defects are upstream and not dependent on SQST-1/p62 in *C. elegans*. The loss of autophagy in conjunction with a functional selective autophagy receptor, such as SQST-1/p62, may exacerbate the disease by further challenging proteostasis (Baskoylu *et al*. 2022).

## Autophagy in longevity

Several models of longevity have been described in *C. elegans*, including mutants in the *daf-2* Insulin IGF-1-like receptor (IIR) or the class I PI3 K *age-1* gene mutants (Kenyon *et al*. 1993), germline-less *glp-1/Notch* mutants (Hsin and Kenyon 1999), dietary-restricted animals (Raizen *et al*. 1995), reduced protein synthesis mutants (Hansen *et al*. 2007), and animals with reduced electron transport function (Feng *et al*. 2001; Dillin *et al*. 2002a; Lee *et al*. 2003) (see Fig. 7).

Autophagy was first demonstrated to be required for the longevity of insulin/IGF-1 *daf-2/IIR* mutants (Meléndez *et al*. 2003). In these initial experiments, RNAi depletion of *bec-1/BECN1* significantly decreased the longevity of *daf-2/IIR* mutant animals, whereas it had almost no effect on wild-type animals. Since then, all longevity models have been found to increase the levels of autophagy, compared to wild-type animals, suggesting that increased autophagy is required for animals to show the lifespan extension (Meléndez *et al*. 2003; Hars *et al*. 2007; Toth *et al*. 2007; Hansen *et al*. 2008; Lapierre *et al*. 2011, 2013). More importantly, autophagy has been confirmed to be indispensable for all longevity models tested in *C. elegans* (Hars *et al*. 2007; Jia and Levine 2007; Tóth *et al*. 2008; Hansen *et al*. 2008; Tang *et al*. 2008; Alvers *et al*. 2009; Bjedov *et al*. 2010; Lapierre *et al*. 2011, 2012; Ruckenstuhl *et al*. 2014) (see Table 2 and Fig. 6). Evidence supporting a beneficial role for autophagy in prolonging lifespan has also been reported in other model organisms, including Drosophila, mouse, and yeast (Hansen *et al*. 2018; Kaushik *et al*. 2021).

**Table 2.** Autophagy genes act in longevity pathways.

Protein	Function/Step	Effect on Longevity
UNC-51	Kinase/Induction	Mutations in the gene (all tissues and whole life) result in faster aging; required for longevity under conditions with mTOR suppression, overexpression of AMPK, rapamycin treatment, germline-less animals and dietary restriction (Tóth *et al*. 2008; Lapierre *et al*. 2011).
ATG-4	Protease/Completion	Required for longevity of *mir-34* loss-of-function mutants (Yang *et al*. 2013a).
BEC-1	Part of the Class III PI3K/Nucleation	Mutations in the gene (all tissues and whole life) result in faster aging; required for longevity under conditions with mTOR suppression and longevity in germline-less animals (Hars *et al*. 2007; Jia and Levine 2007; Toth *et al*. 2007; Hansen *et al*. 2008; Meléndez *et al*. 2008; Eisenberg *et al*. 2009; Lapierre *et al*. 2011, 2013; Yang *et al*. 2013a; Ryu *et al*. 2016).
VPS-34	Class III PI3K/PIK3C Nucleation	Required for longevity under dietary restriction, longevity in germline-less animals and after urolithin A treatment (Jia and Levine 2007; Hansen *et al*. 2008; Lapierre *et al*. 2011; Ryu *et al*. 2016).
ATG-7	E1 enzyme/Completion	RNAi during adulthood decreases lifespan; required for longevity under spermidine treatment and dietary restriction (Hars *et al*. 2007; Jia *et al*. 2007; Eisenberg *et al*. 2009).
ATG-9	Transmembrane protein/Phagophore Formation	Required for longevity of *mir-34* loss-of-function mutants (Yang *et al*. 2013a).
LGG-3/ATG12	Protein that forms a complex with ATG-5 and ATG-16.2/Completion	Required for longevity under insulin IGF-1 inhibition, or dietary restriction (Hars *et al*. 2007).
ATG-18	PtdIns3P-binding protein/Formation	Required for longevity under insulin IGF-1 inhibition, mTOR suppression, and dietary restriction, lack of germline, reduced mitochondrial respiration, inhibition of S6K, or AMPK overexpression (Toth *et al*. 2007; Lapierre *et al*. 2011; Gelino *et al*. 2016; McQuary *et al*. 2016; Chang *et al*. 2017; Minnerly *et al*. 2017).
PINK-1	Mitochondrial kinase/Mitophagy	Required for longevity under insulin IGF-1 inhibition, conditions after urolithin A treatment and NAM riboside treatment (Palikaras *et al*. 2015; Schiavi *et al*. 2015; Ryu *et al*. 2016).
SQST-1/p62	Ubiquitin binding protein/Selective Autophagy Receptor	Required for longevity under conditions after urolithin A (Schiavi *et al*. 2015; Ryu *et al*. 2016; Kumsta *et al*. 2019).
HLH-30/TFEB	Transcription Factor/Induces autophagy	Required for longevity under conditions of mTOR inhibition, dietary restriction, inhibition of insulin IGF-1 signaling, inhibition of S6K, lack of germ line, reduced mitochondrial respiration; overexpression results in autophagy dependent increased lifespan (Lapierre *et al*. 2013; McQuary *et al*. 2016).

## Regulation of autophagy for longevity

Several transcription factors have been shown to promote autophagy at the level of gene expression in response to different environmental stressors, including DAF-16/FOXO (Lin *et al*. 1997), PHA-4/FOXA (Hansen *et al*. 2008), HLH-30/TFEB (Lapierre *et al*. 2013; O'Rourke and Ruvkun 2013), SKN-1/Nrf2 (Tullet *et al*. 2008), HSF-1 (Kumsta *et al*. 2017), MML-1/MXL-2 or Mondo/Mlx (Nakamura *et al*. 2016), and nuclear hormone receptors, such as NHR-62, the HNF4-related hormone receptor (Heestand *et al*. 2013).

TFEB is a master transcription factor for the autophagy-lysosomal pathway (Settembre *et al*. 2008). Upon autophagy induction, TFEB shuttles from the cytoplasm into the nucleus, a process controlled by nutrient-responsive phosphorylation of TFEB (Puertollano *et al*. 2018). The *C. elegans* TFEB homolog HLH-30 also translocates into the nucleus upon starvation, and activates the expression of autophagy genes (Lapierre *et al*. 2013; O'Rourke and Ruvkun 2013; Settembre *et al*. 2013a). Under nutrient-rich conditions, mTOR (in *C. elegans*LET-363/mTOR) phosphorylates HLH-30/TFEB at the lysosomal surface, promoting its cytosolic retention bound to 14-3-3 and thereby preventing its translocation to the nucleus (Peña-Llopis *et al*. 2011; Settembre *et al*. 2011, 2012; Martina *et al*. 2012; Roczniak-Ferguson *et al*. 2012). Inhibition of mTOR, e.g. by nutrient deprivation or by RNAi depletion, results in localization of HLH-30/TFEB to the nucleus to coordinate the expression of autophagy genes acting in all stages of the process (Lapierre *et al*. 2013; O'Rourke and Ruvkun 2013). Similarly, nuclear translocation of HLH-30/TFEB is observed upon removal of the germ line, in animals with defects in *daf-2/IIR* signaling, reduced mRNA translation, or reduced mitochondrial respiration, and all these longevity programs require HLH-30/TFEB to extend lifespan (Lapierre *et al*. 2013). Moreover, overexpression of HLH-30/TFEB activates autophagy and moderately extends lifespan (Lapierre *et al*. 2013). TFEB-mediated transcriptional induction of autophagy may be important for promoting autophagic flux and providing a pool of metabolites, particularly lipids, to maintain homeostasis and prolong lifespan. The Myc superfamily basic helix–loop–helix (bHLH) transcription factor MML-1 (Myc and Mondo-like 1) and its heterodimer partner MXL-2 (MAX-like 2) were identified as modulators of the longevity conferred to germline-less animals (Nakamura *et al*. 2016). The Mondo family transcription factor MXL-3 protein (a paralog of MXL-1 (Max)), antagonizes the activity of HLH-30 to repress lipase genes in nutrient-rich conditions (O'Rourke and Ruvkun 2013). MXL-1/MXL-2 belongs to the Myc and Mondo family and their homologs, MondoA/MLX or ChREBP/MXL function as a glucose sensor (Sans *et al*. 2006; Havula and Hietakangas 2012). The HIPK homeodomain-interacting protein kinase (HPK-1) also acts to induce autophagy in response to dietary restriction or inactivation of TORC1 (Samuelson *et al*. 2007; Das *et al*. 2017; Lazaro-Pena *et al*. 2023). HPK-1 requires the Myc-family of transcription factors to induce autophagy, preserve neuronal integrity, improve proteostasis, and extend lifespan. Thus, animals can cope with nutrient fluctuation by accurately sensing food availability and rapidly adjusting their metabolism.

There are several models of dietary restriction in *C. elegans* (Greer and Brunet 2009). *eat-2* mutants have a mutation in an acetylcholine receptor that causes defective pharyngeal pumping, reduced food intake, and long-lived phenotype. *eat-2* mutants display an increase in GFP::LGG-1 positive foci, and require autophagy genes (*unc-51/ULK, bec-1/Becn1, vps-34*, *atg-18*, and *atg-7/Atg7*) for the long-lived phenotype (Jia and Levine 2007; Hansen *et al*. 2008). Dietary-restricted animals require HLH-30/TFEB and PHA-4/FOXA for longevity (Hansen *et al*. 2008; Heestand *et al*. 2013; Lapierre *et al*. 2013). The longevity induced by dietary restriction can be partly mediated via TOR, since TOR inhibition of *eat-2* mutants does not further extend lifespan (Hansen *et al*. 2008). The lifespan resulting from reduced TOR requires PHA-4/FOXA and HLH-30/TFEB activity (Sheaffer *et al*. 2008; Lapierre *et al*. 2013). It has been reported that in response to dietary restriction, the activities of HLH-30/TFEB and PHA-4/FOXA are coordinately regulated through an epigenetic mechanism that involves the activity of SAMS-1 and histone methylation (Lim *et al*. 2023). Dietary restriction represses SET-2, a histone H3K4 methyltransferase, and reduced the availability of S-adenosyl methionine and H3K4me3 levels, which in turn activate HLH-30/TFEB and PHA-4/FOXA (Lim *et al*. 2023). Germline-less mutants result from either ablation of the cells that will give rise to the germ line, or a mutation in *glp-1/Notch* (Hsin and Kenyon 1999; Wang *et al*. 2008). Germline-less mutants require HLH-30/TFEB, MML-1/MXL-2, and PHA-4 to induce autophagy (Lapierre *et al*. 2011, 2013; Nakamura *et al*. 2016).

## Autophagy levels decline with age

There is considerable evidence for a decline in autophagic degradation with age in several model systems, which may result in the accumulation of dysfunctional organelles and damaged proteins that in turn contribute to cellular aging (Sarkis *et al*. 1988; Cuervo and Dice 2000; Del Roso *et al*. 2003; Sun *et al*. 2020). The construction of a complete single-cell gene expression atlas in *C. elegans* demonstrated that there are coordinated changes in cell-type-specific function, with the downregulation of energy metabolism (Roux *et al*. 2023). To monitor autophagy, flux assays have been carried out with a dual fluorescently tagged LGG-1 protein containing GFP and mCherry, as described above, section “Monitoring autophagy in *C. elegans*” (Chang *et al*. 2017). This dual fluorescent LGG-1, together with autophagy inhibitors, showed an age-dependent increase in the number of autophagosomes and autolysosomes in body wall muscle, intestine, pharyngeal muscle, and neurons. Similarly, autophagic flux was also reported to be diminished in whole body extracts of aged animals (Wilhelm *et al*. 2017). Collectively, these results suggest compromised autophagic activity with age (Chang *et al*. 2017). Of note, autophagy has also been consistently found to be dysfunctional in age-related disorders, such as neurodegenerative diseases, cancer, or diabetes (Hara *et al*. 2006; Menzies *et al*. 2015; Levy *et al*. 2017; Zhao and Zhang 2019b; Debnath *et al*. 2023). Autophagy may act to recycle unnecessary or damaged macromolecules and organelles to provide raw material for new synthesis to rejuvenate a cell. However, what is being degraded, and in what tissues autophagy is required, are questions that are still far from being resolved.

Overexpression of autophagy genes in *C. elegans* does not result in a major effect on longevity, but has been reported to increase lifespan in other organisms, such as Drosophila, by promoting basal levels of autophagy in the nervous system or in muscle (Simonsen *et al*. 2008; Ulgherait *et al*. 2014). The only exception is that overexpression of HLH-30/TFEB was shown to extend lifespan in an autophagy-dependent manner (Lapierre *et al*. 2013). Treatment of TFEB agonists have been shown to extend lifespan (Wang *et al*. 2017). Overexpression of the autophagy adaptor SQST-1/p62 was also found to promote proteostasis, induce autophagy and increase lifespan in *C. elegans* (Kumsta *et al*. 2019), but these results have been controversial, as several overexpressing SQST-1/p62 strains were found not to have an extended lifespan in a recent publication (Kumar *et al*. 2023). The reasons for this difference in the results are not clear. Suppression of Rubicon, a negative regulator of autophagy, was also found to promote longevity (Nakamura *et al*. 2019), and the expression of Rubicon increases with age, suggesting that this is one way in which autophagy may decrease during aging.

The MML-1/MXL-2 family of transcription factors was shown to be involved in longevity (Johnson *et al*. 2014; Nakamura *et al*. 2016). MML-1/MXL-2 belongs to the Myc and Mondo family member and is required for the longevity conferred by reduced TOR signaling, reduced Insulin IGF-1 signaling, and reduced mitochondrial respiration (Johnson *et al*. 2014; Nakamura *et al*. 2016). Inhibition of MML-1/MXL-2 was shown to impair HLH-30 nuclear localization and the activation of autophagy.

MondoA is a transcription factor that partners with Max-like protein X (Mxl) and its decline during aging was found to regulate cellular senescence, autophagy, and mitochondrial homeostasis (Yamamoto-Imoto *et al*. 2022). MondoA acts in a complex that promotes longevity in response to germ line removal or dietary restriction (Johnson *et al*. 2014; Nakamura *et al*. 2016). In addition, depletion of either *mml-1* (homolog to MondaA/ChREBP) or *mxl-2* (homolog to MLX) abrogated the induction of autophagy that normally occurs in *glp-1/Notch* germline-less mutants (Nakamura *et al*. 2016). Similarly, MML-1/MXL-2 is required for the longevity of animals with reduced insulin/IGF-1 signaling, reduced TOR or reduced mitochondrial respiration. Transcriptome analysis found that MML-1/MXL-2 and HLH-30 have many shared target genes including lysosomal genes, but also have preferential targets, for example, *atg-2/ATG2*, *atg-9/ATG9,* and *epg-9/ATG101* are preferentially regulated by MML-1/MXL-2, whereas *unc-51/ULK* and *lgg-1/LC3* are regulated by HLH-30 (Nakamura *et al*. 2016). The spatial and temporal regulation of autophagy, the transcription factors required and how their regulation (time and place) of autophagy may vary under the different longevity models and needs to be further investigated.

## Where is autophagy required for longevity?

Clearly, autophagy acts cell nonautonomously to improve the state of other tissues. To understand where autophagic degradation is required for longevity, spatiotemporal analysis of autophagy reporters was carried out using a tandem-tagged mCherry-GFP-Atg8 reporter, combined with inhibition by Bafilomycin to stop simultaneously flux and monitor the formation of autophagosomes and their degradation (Chang *et al*. 2017). These spatiotemporal studies of autophagic flux (the capacity to degrade by autophagosomes) in animals of various ages indicated that aging of wild-type animals was associated with an accumulation of stalled autophagic vesicles, and an age-dependent decrease in autophagic flux in many tissues, including the intestine, body-wall muscle, the pharynx, and nerve ring neurons (Chang *et al*. 2017, 2020). These studies further suggest a systemic, progressive decline in autophagic degradation capacity, referred to as autophagic flux (Chang *et al*. 2017).

The decline of autophagy may be systematically controlled with aging, where tissues cross-talk with each other to coordinate levels of autophagic degradation. To this effect, upregulation of a secreted microRNA, *mir-83/mir-29,* was found to act in the intestine of aging animals to impair autophagy via the inhibition of the *mir-83* target CUP-5/MCOLN (Zhou *et al*. 2019). The *mir-83*-driven regulation was found to occur in the intestine and body wall muscle. However, no expression of *mir-83* has been documented in body wall muscle (Martinez *et al*. 2008; Burke *et al*. 2015; Zhou *et al*. 2019). This observation together with detection of *mir-83* in purified exosomes, drove Zhou *et al*. (2019) to speculate that *mir-83* is transported from the intestine into body wall muscle via exosomes in pseudocoelomic fluids.

Analysis of autophagic flux in long-lived *daf-2*/insulin IGF-1 signaling mutants and the *glp-1/Notch* receptor mutants, revealed that autophagy was differentially regulated, both spatially and temporally. Germline-less *glp-1/Notch* animals, but not *daf-2/IIR* mutants, require autophagy gene activity in the intestine for the lifespan extension (Chang *et al*. 2017). RNAi depletion of intestinal *atg-18/Wipi2* had no significant decrease in the lifespan of *daf-2/IIR* mutants, despite the fact that *daf-2/IIR* mutants show an improved intestinal barrier function (Gelino *et al*. 2016). Therefore, in *daf-2/IIR* long-lived mutants, intestinal autophagy may not be required.

Intestine-specific expression of autophagy genes appears to be also required for the lifespan extension of dietary restriction in *C. elegans* (Gelino *et al*. 2016). Various reporters of autophagic activity and flux analyses indicate that autophagy is induced in the intestine of long-lived *eat*-*2* mutants. Furthermore, intestine-specific RNAi of two LC3/GABARAP family homologs, *lgg*-*1* and *lgg*-*2* or of the *WIPI* homolog *atg*-*18* significantly abrogated the extended lifespan of *eat-2* mutant worms (Gelino *et al*. 2016). Thus, intestinal autophagy is required for the longevity mediated by dietary restriction. Collectively, these studies show that the spatial requirement for autophagy may be different in different models of longevity.

## Selective forms of autophagy in longevity

Selective forms of autophagy have also been implicated in longevity, suggesting an additional level of specificity and regulation. Selective autophagy pathways include mitophagy (the degradation of mitochondria), lipophagy (the degradation of lipids), aggrephagy (the degradation of aggregates), and lysophagy (degradation of lysosomes). Regardless, in every case, autophagy is required for the observed extension of lifespan. It is difficult to separate the action of genes that function in the bulk autophagy pathway from those that act in selected forms of autophagy, and this is probably the most important question that needs to be investigated in the future for any potential requirement for autophagy.

Below are examples of selected forms of autophagy that have been shown to influence aging.

### Aggrephagy

Age-dependent proteotoxicity can be modeled in *C. elegans*, for example Huntington's disease is caused by the presence of a polyglutamine (Poly Q) expansion in the protein huntingtin, which renders it prone to aggregation (Hsu *et al*. 2003; Morley and Morimoto 2004; Ben-Zvi *et al*. 2009). Aggregation of the Poly Q-containing proteins can be assayed and usually begins at the onset of adulthood (Hsu *et al*. 2003; Ben-Zvi *et al*. 2009). The proteostatic network declines with age, but this decline can be suppressed by the overexpression of HSF-1 (Morley *et al*. 2002; Hsu *et al*. 2003; Morley and Morimoto 2004), which increases longevity and improves stress resistance. In hormesis, the overexpression of HSF-1 results in a long lifespan, resistance to heat stress, and a decrease in several models of aggregation (Kumsta *et al*. 2017), see Hormesis below.

### Lysophagy

Lysosomes can act as major signaling hubs and can sense and response to metabolic shifts in the cell, as they maintain cellular homeostasis (Settembre *et al*. 2013b). Maintenance of lysosomal integrity is important, and the lysosomal membrane contains heavily glycosylated membrane proteins that appear to form a continuous carbohydrate layer at the luminal leaflet to prevent the leakage of any acid hydrolases (Fukuda 1991; Saftig *et al*. 2010). The lysosomal glycoproteins include LAMP-1 (in *C. elegans*LMP-1), LAMP-2 (in *C. elegans*LMP-2), and the lysosomal integral membrane proteins LIMP-1/CD63 and LIMP-2. Loss of lysosomal integrity can trigger lysophagy (Raben *et al*. 2007), or the selective degradation of lysosomes. For a review of lysophagy, see Papadopoulos *et al*. (2020). The *C. elegans* protein SCAV-3, the ortholog of LIMP-2, was reported to act as a regulator of lysosome membrane integrity (Li *et al*. 2016). Loss of *scav-3* resulted in ruptured lysosomes and a shortened lifespan (Li *et al*. 2016), phenotypes that can be suppressed by overexpression of LMP-1 or LMP-2, the *C. elegans* LAMPs, suggesting that longevity requires lysosomal integrity. In addition, a reduction in DAF-2/IIS suppressed the lysosomal damage and extended the lifespan of *scav-3* mutants, in a DAF-16-dependent manner. Thus, lysosome integrity can be modified by the insulin IGF-1 signaling pathway to promote longevity (Li *et al*. 2016). Moreover, the inhibition of lysosomal damage observed in *scav-3* mutants that also overexpress LMP-1 or LMP-2, did not require DAF-16/FOXO, suggesting that the effect of LMP-1 and LMP-2 was independent of DAF-16/FOXO. Interestingly, the appearance of GFP::Gal3 positive (reporter for damaged lysosomes) was unchanged in autophagy defective mutants that also carried the *scav-3* mutation, when compared with *scav-3* single mutants. Thus, the loss of SCAV-3 may affect the removal of lysosomes by autophagy or alternatively there are autophagy-independent mechanisms involved (Li *et al*. 2016).

### Mitophagy

In *C. elegans*, mitophagy, the selected targeting of mitochondria for degradation by autophagy, cooperates with mitochondrial biogenesis to regulate mitochondrial content and longevity (Palikaras *et al*. 2015). DCT-1 (DAF-16/FOXO Controlled, germline Tumor affecting-1) (Palikaras *et al*. 2015) is an ortholog of the mammalian NIX/BIP3L and BNIP3 (Nip3-like protein X/Bcl-2 and adenovirus E1B interacting protein), a mitophagy receptor in mammals (Schweers *et al*. 2007; Sandoval *et al*. 2008; Zhang *et al*. 2012), which acts together with PINK-1 (PTEN-induced putative kinase protein 1) and PDR-1/Parkin (a E3 ubiquitin ligase), as key mediators of mitophagy (Palikaras *et al*. 2018; Pickles *et al*. 2018; Ng *et al*. 2021). When damaged mitochondria causes membrane depolarization, impaired import of PINK1 results in its stabilization on the outer mitochondrial membrane, which recruits Parkin, which subsequently ubiquitylates proteins. The ubiquitin motifs are recognized by a set of autophagy receptor proteins that interacts with an LC3-interacting region (LIR) motif that connects to the IM, enabling the engulfment of mitochondria by the autophagosome (Palikaras *et al*. 2018; Pickles *et al*. 2018; Ng *et al*. 2021).

Mitophagy was induced in *daf-2/IIR* long-lived mutants, and compromising mitophagy by knockdown of *dct-1*, *pink-1*, and *pdr-1/Parkin* significantly shortened the lifespan of *daf-2/IIR* mutants (Palikaras *et al*. 2015). Similarly, *dct-1* and *pink-1* were also required for the longevity of mitochondrial mutants *isp-1* or *clk-1* and for the dietary-restricted *eat-2* mutants (Palikaras *et al*. 2015). For these experiments, a mitochondria-targeted Rosella (mtRosella) biosensor, which combines a pH-insensitive DsRed fused to the pH-sensitive GFP, was employed. The GFP part of the biosensor is quenched in the acidic autolysosome, while the DsRed fluorophore is resistant. In transgenic animals with the mtRosella biosensor, stimulation of mitophagy was noted by the reduction in GFP/DsRed ratio (Palikaras *et al*. 2015). In parallel, transgenic animals expressing a mitochondria-targeted GFP and the DsRed::LGG-1 autophagosomal marker was used to detect mitophagy in body wall muscles (Palikaras *et al*. 2015). *dct-1*, *pdr-1*, and *pink-1* mutants are more sensitive to stressors, such as ultraviolet radiation, paraquat treatment, or starvation, and DCT-1 overexpression was found to allow animals to better adapt to stress in a PINK-1 and PDR-1-dependent manner (Palikaras *et al*. 2015). In mitophagy gene-depleted animals, decreased ATP levels, accumulated ROS, mitochondrial membrane depolarization, increased oxygen consumption, and elevated cytoplasmic Ca^2+^ levels were found to be worse under stress conditions, all suggestive of mitochondrial dysfunction (Palikaras *et al*. 2015). Sustained intracellular Ca^2+^ signal triggers opening of the mitochondrial permeability transition pore (mPTP), mitochondrial depolarization, and activation of the Ca^2+^-dependent phosphatase calcineurin A, which dephosphorylates DRP-1 to drive mitochondrial fragmentation, a prerequisite for mitophagy (Augustine *et al*. 2003; Rizzuto *et al*. 2012; Brini *et al*. 2014; Bhosale *et al*. 2015; Giorgio *et al*. 2018). Localized elevation of Ca^2+^ activates mitophagy through the interplay of AMPK and calcineurin A in motor neurons (Zaninello *et al*. 2022). The chelation of Ca^2+^ or inhibition of calcineurin A protects GABAergic neurons from death and from mitochondrial dysfunction (Zaninello *et al*. 2022). Thus, Ca^2+^-sustained levels activate calcineurin A and AMPK to regulate mitochondrial dynamics and neuronal mitophagy (Zaninello *et al*. 2022).

SKN-1/NRF2 (nuclear factor-erythroid 2-related factor) was found to be a key player that couples mitochondrial biogenesis and mitophagy (Palikaras *et al*. 2015). SKN-1 is activated under oxidative stress (Ghose *et al*. 2013; Ploumi *et al*. 2017), and promotes mitochondrial biogenesis by upregulating the expression of several mitochondrial genes. SKN-1 was found to be required for the expression of *dct-1* and *daf-16* and its depletion disrupted DCT-1 mediated mitophagy, decreased mtDNA content, caused mitochondrial membrane depolarization, and increased cytoplasmic Ca^2+^ (Palikaras *et al*. 2015). Thus, biogenesis and turnover of mitochondria are coordinated to properly respond to energy demands, and stress.

In a screen for RNA-binding proteins that are altered upon aging, the RNA-binding protein PUF-8/PUM2, a translation repressor was found to regulate mitophagy (D'Amico *et al*. 2019). RNA-binding proteins have low-complexity domains that mediate their condensation in ribonucleoproteingranules and control mRNA translation, metabolism, and transport (Sheinberger and Shav-Tal 2017). In response to stress, RNA-binding proteins and their target mRNAs assemble in stress granules (Sheinberger and Shav-Tal 2017). The interaction of two mRNAs, *puf-8* and *mff-1* RNAs, was found to regulate mitophagy (D'Amico *et al*. 2019). The product of the *mff-1* gene, the mitochondrial fission factor MFF-1, is an outer membrane mitochondrial protein that recruits the mitochondrial fission factor DRP-1 to mitochondria (Otera *et al*. 2010). In aged animals, the *puf-8* mRNA regulates mitophagy by repressing the translation of the *mff-1* mRNA, resulting in a decrease in mitochondrial fission and the accumulation of altered fragmented mitochondria (D'Amico *et al*. 2019). *puf-8* knockdown in old *C. elegans*, and CRISPR/Cas9-mediated knockout of its ortholog *Pum2* in elderly mice, enhanced mitochondrial fission and mitophagy (D'Amico *et al*. 2019).

Frataxin (FRH-1) is a nuclear-encoded mitochondrial protein involved in the biogenesis of iron–sulfur (Fe–S)-cluster proteins and iron homeostasis. The human gene is involved in Friedreich's ataxia, a devastating and progressive neurodegenerative disorder characterized by impaired coordination and muscle weakness (Foury *et al*. 2007; Anzovino *et al*. 2014). Partial depletion of *frh-1* increases autophagy and extends the lifespan of wild-type animals (Schiavi *et al*. 2015), although other reports have found it to shorten lifespan (Vázquez-Manrique *et al*. 2006; Zarse *et al*. 2007). The reasons for these conflicting results remain unknown, although it has been proposed that experimental differences regarding the application of RNAi (microinjection vs feeding) and different RNAi constructs could be the source of the conflict (Ventura *et al*. 2006). The depletion of FRH-1 protein expression has been found to be part of an iron starvation response that extends lifespan and increases mitophagy in a HIF-1-dependent manner (Schiavi *et al*. 2013, 2015). Similar to the pro-longevity effects of a partial depletion of *frh-1*, nontoxic levels of iron depletion with iron chelators, such as bypiridine, or the hypoxia mimetic CoCl_2_, extend lifespan through mitophagy (Schiavi *et al*. 2022, 2023). Exposure to nonlethal levels of hypoxia during development, referred to as hypoxia preconditioning, prevents the detrimental effects of severe hypoxia-induced neurodegeneration later in life (Dasgupta *et al*. 2007; Liu *et al*. 2021a). Thus, the antiaging effects of *frh-1* depletion are proposed as a potential strategy to delay aging and slow age-associated neuromuscular pathologies (Schiavi *et al*. 2023). Gene expression analysis found changes in gene expression for genes that regulate mitochondrial activities, carbon and lipid metabolism, as well as genes involved in redox homeostasis in animals with partial depletion of *frh-1* (Schiavi *et al*. 2023). As a beneficial role of glutathione-regulated pathways, loss of glutathione redox homeostasis impairs proteostasis by inhibiting autophagy in *C. elegans* neurodegenerative disease models (Guerrero-Gómez *et al*. 2019). Similar to mitochondrial stress (Dillin *et al*. 2002b; Rea *et al*. 2007), which has to be applied during development for its pro-longevity effects, the metabolic remodeling associated with mitochondrial hormesis and/or iron depletion must occur early in life to promote longevity (Schiavi *et al*. 2023). *frh-1* RNAi depletion in *C. elegans* increases the pool of reduced glutathione and decreases iron ROS and lipid content, suggesting that frataxin depletion suppresses ferroptosis (Schiavi *et al*. 2013, 2015, 2023). Whether the reduction in iron availability conferred by iron chelators, and lipid remodeling impact ferroptosis through the same mechanisms, and if autophagy and ferroptosis cross talk to regulate health span and longevity under iron and/or FRH-1/frataxin depletion, are still questions that remain.

CISD-1, the nematode ortholog for mammalian mitoNEET and CISD2, is a mitochondrial iron–sulfur cluster binding protein that acts to maintain iron homeostasis and may provide a model to study the Wolfram neurodegenerative syndrome and related diseases (Hsiung *et al*. 2020; Ploumi *et al*. 2023). CISD-1 modulates longevity by engaging both autophagy and the mitochondrial intrinsic apoptosis pathway (Ploumi *et al*. 2023). Loss of proapoptotic CED-3, CED-4, and CED-13 or gain-of-function mutations in the antiapoptotic CED-9 were found to act downstream of CISD-1 to maintain neuronal integrity, mitochondrial bioenergetics, and promote lifespan (Ploumi *et al*. 2023). Intracellular levels of iron appeared to be important for CISD-1 function, as mild iron supplementation slowed down aging and improved the impaired mitochondrial energy production in animals lacking CISD-1 (Ploumi *et al*. 2023). In contrast, limiting iron availability at sublethal doses with an iron chelator, or through *frh-1* silencing, protects against hypoxia, delays functional decline and extends lifespan through a hypoxia-like induction of mitophagy that protects against age-induced proteotoxicity (Schiavi *et al*. 2023). It has been proposed that the beneficial effects of silencing *frh-1* are in part mediated by counteracting ferroptosis, mediated by iron-induced lipid (polyunsaturated fatty acids) peroxidation (Schiavi *et al*. 2023). As Schiavi *et al*. highlights, it may be difficult to figure out the oxygen levels or mitochondrial activity that trigger the beneficial effects of hormesis. However, interventions that mimic hypoxia or mitochondria preconditioning may be more feasible as preventive or therapeutic approaches to treat neuronal pathologies associated with aging decline (Schiavi *et al*. 2023). The beneficial effect of iron depleting agents is dose-dependent, and its associated metabolic remodeling must occur early in life to promote protection against aging and age-associated features (Maglioni *et al*. 2014).

Recent studies have found that a subset of SNARE proteins (soluble N-ethylmaleimide-sensitive factor attachment protein receptors) localize to or in the vicinity of mitochondria, referred to as mitoSNARE factors (Gkikas *et al*. 2023). SNARE proteins are involved in the fusion of membranes including that of organelles and the plasma membranes, t-SNAREs for target membranes, and v-SNAREs for vesicle SNAREs (Yoon and Munson 2018; Sauvola and Littleton 2021). The mitoSNARE proteins include the t-SNARE syntaxin SYX-17, the v-SNAREs synaptobrevins VAMP-7 and SNB-6, as well as the tethering factor USO-1, which were found to regulate mitochondrial abundance and basal levels of autophagy (Gkikas *et al*. 2023). RNAi inhibition of the SNARE disassembly gene *nsf-1* reduced mitochondrial mass and NSF-1 activity was required for the phenotypes associated with loss of mitoSNARE activity (Gkikas *et al*. 2023). Finally, the mitoSNAREs were found to be required in neuronal and nonneuronal tissues for normal aging (Gkikas *et al*. 2023). The detrimental effects of mitoSNARE gene depletion on aging suggest that these proteins play a role in basal autophagy regulation and aging.

Heteroplasmy of mitochondria DNA (mtDNA), the presence of more than one mitochondrial genome, is considered a hallmark of aging (Michikawa *et al*. 1999). Homogeneity of mtDNA occurs by the selective removal of deleterious mtDNA in the female germ line in Drosophila (Lieber *et al*. 2019). Homogeneity of mtDNA also occurs by the removal of paternal mitochondria after fertilization in *C. elegans*, Drosophila, and mouse (see section “Clearance of paternal mitochondria and Mos”) (Al Rawi *et al*. 2011; Sato and Sato 2011; Politi *et al*. 2014; Rojansky *et al*. 2016; Sato *et al*. 2018). These processes have been shown to require mitophagy. In *C. elegans*, the elimination of paternal mitochondria has been extensively studied, and a delay in clearance after fertilization leads to embryonic lethality (Zhou *et al*. 2016). However, the mechanisms involved for the role of mtDNA number variation in aging and associated diseases are not well understood.

### Lipophagy

Recent studies in *C. elegans* have shown a connection between lipid metabolism and lifespan. Alterations in lipid metabolism have been associated with several long-lived mutants, as for example *glp-1/Notch* germline-less and *daf-2/II* mutants (Kimura *et al*. 1997; Ashrafi *et al*. 2003; Perez and Van Gilst 2008; Wang *et al*. 2008; O'Rourke *et al*. 2009). Lifespan extension in *daf-2/IIR* or germline-less *glp-1/Notch* mutants display an increase in GFP::LGG-1-positive foci in seam cells and require autophagy gene activity (Meléndez *et al*. 2003; Lapierre *et al*. 2011). *glp-1/Notch* mutants also require the lysosomal acid lipase LIPL-4 activity, which is specifically expressed in the intestine (Wang *et al*. 2008). LIPL-4 is upregulated upon fasting and is also upregulated in long-lived mutants with reduced insulin IGF-1 signaling (Wang *et al*. 2008; Mony *et al*. 2021). Autophagy-dependent lipolysis was found to promote longevity independent from LIPL-4 in *glp-1/Notch* mutants (Lapierre *et al*. 2011). Overexpression of LIPL-4 induces the nuclear translocation of the lipid chaperone lipid-binding protein 8 (LBP-8) and promotes longevity by activating the nuclear hormone receptor 49 (NHR-49) (Folick *et al*. 2015). More recently, peripheral lysosomal lipolysis resulting from constitutively expressing *lipl-4* in the intestine was found to upregulate neuropeptide signaling in the nervous system that promotes longevity (Savini *et al*. 2022). This cell nonautonomous regulation is mediated by dihomo-γ-linolenic acid, and LBP-3, a lipid chaperone protein, which act though the NHR-49 nuclear receptor and NLP-11 neuropeptide in neurons to extend lifespan. Thus, lysosomes act as a signaling hub that coordinates metabolism and aging (Savini *et al*. 2022).

An important process that facilitates lipid transport between tissues is associated with longevity, and overexpression of the yolk lipoprotein vitellogenin reduced the lifespan of *daf-2/IIR* or *glp-1/Notch* mutant animals by impairing the induction of autophagy and lysosomal genes (Seah *et al*. 2016). In contrast, *vit* gene silencing enhanced the activity of PHA-4/FOXA and DAF-16/FOXO and induced the expression of autophagy and lysosomal acid lipase genes, resulting in an extended lifespan (Seah *et al*. 2016). *vit* gene silencing failed to extend the lifespan of *daf-16/FOXO* and *hlh-30/TFEB* mutants, indicating a role for these transcription factors in the longevity of *vit* gene silenced animals (Seah *et al*. 2016). The longevity of *vit* gene silenced animals also required the NHR-49 and NHR-80, thus, the regulation of yolk lipoprotein biogenesis can modulate aging by affecting the transcriptional activation of autophagy and lysosomal lipolytic genes (Seah *et al*. 2016).

## Heat shock or pharmacological treatments that induce autophagy and prolong lifespan

Several treatments have been shown to prolong lifespan, including hormetic stress, and the treatment with spermidine, urolithin A, metformin, rapamycin, or nicotinamide-adenine dinucleotide (NAD)+. Even if the exact mechanism of action for these treatments is not known, they have all been found to induce some form of autophagy. Again, the tissues, the mechanisms and the types of autophagy, bulk, or selective autophagy, are aspects that have yet to be elucidated (see Fig. 7).

### Hormesis

Hormetic stress results from the exposure to an external stressor that is toxic at high doses, but beneficial at lower doses (Lithgow *et al*. 1995; Gems and Partridge 2008; Rattan 2008). This phenomenon has been found to work in several species, including *C. elegans*, Drosophila and human fibroblasts (Khazaeli *et al*. 1997; Le Bourg *et al*. 2000; Butov *et al*. 2001; Fonager *et al*. 2002; Hercus *et al*. 2003; Kristensen *et al*. 2003; Rattan and Ali 2007; Kumsta *et al*. 2017). A mild heat shock early in life has been shown to result in a beneficial treatment that promotes fitness, lifespan extension and a reduction in protein aggregation (Kumsta *et al*. 2017). Overexpression of the conserved transcription factor acting in the heat-shock response HSF-1 can induce a hormetic response, which improves proteostasis, increases longevity and improves stress resistance in *C. elegans* (Kumsta *et al*. 2017). Both heat shock and overexpression of HSF-1 induce autophagy in multiple tissues and autophagy genes were found to be essential for their stress resistance and longevity (Kumsta *et al*. 2017). Hormetic shock also increased proteostasis that improved several models of protein aggregation in an autophagy-dependent fashion (Kumsta *et al*. 2017). Thus, autophagy induction by hormetic heat stress enhances proteostasis and prolongs lifespan. Although autophagy genes are required, the mechanisms by which hormesis act to improve proteostasis, decrease aggregation or prolong lifespan are not clear. Hormesis can also be induced by a mild depletion of different mitochondrial electron transport chain regulatory subunits, mild hypoxia, mild iron depletion, and mild frataxin (FRH-1) depletion (see Mitophagy section) (Schiavi *et al*. 2013, 2015, 2023; Maglioni *et al*. 2014).

### Spermidine treatment requires autophagy for longevity

The administration of spermidine, a natural polyamine, for which intracellular concentrations decline during human aging, results in an extension of lifespan in yeast, Drosophila, *C. elegans*, and human immune cells (Eisenberg *et al*. 2009; Hofer *et al*. 2024). Conversely, depletion of polyamines in yeast resulted in hyperacetylation, ROS generation, early necrotic cell death, and decreased lifespan. The alterations in chromatin acetylation status were shown to upregulate several autophagy genes, triggering autophagy in yeast, Drosophila, *C. elegans*, and human cells. Thus, a model where spermidine inhibits the activity of histone deacetylases, results in the induction of autophagy gene transcription that then in turn prolongs lifespan (Eisenberg *et al*. 2009). In a recent report, spermidine levels were reported to increase under fasting or caloric restriction in various species, including yeast, worms, flies, mice, and human volunteers, whereas disruption of the polyamine pathway abrogated the effects of fasting (Hofer *et al*. 2024). Mechanistically, spermidine is thought to mediate its effects by inducing autophagy and hypusination of the translation regulator eIF5A, in the context of fasting regimens (Hofer *et al*. 2024). Hypusine is an unconventional amino acid, formed by a unique posttranslational modification of a conserved lysine residue of eIF5A. Hypusination is required for the activity of eIF5A, the only known eukaryotic protein to contain hypusine (Park and Wolff 2018).

### Urolithin A increase in lifespan is autophagy dependent

Urolithin A, the endproduct of both ellagitannins and ellagic acid, found in nuts, pomegranate, and berries, extends lifespan and improves fitness during aging (Ryu *et al*. 2016). Lifespan extension following urolithin A treatment was dependent on the expression of autophagy genes *bec-1*, *sqst-1/p62*, and *vps-34*, and the mitophagy genes *pink-1*, *dct-1*, and *skn-1/Nrf2* (Ryu *et al*. 2016). Urolithin A treatment prevented the accumulation of dysfunctional mitochondria, and prolonged normal mobility and pharyngeal pumping, while maintaining respiratory capacity (Ryu *et al*. 2016). Both *daf-16/FOXO* and *eat-2* mutants showed an extended lifespan with urolithin A treatment, while in contrast, life extension by urolithin A treatment was partially dependent on AMPK (Ryu *et al*. 2016). The effect of urolithin A was also dependent on mitochondrial function, as it was completely suppressed in *mev-1* (mitochondrial succinate dehydrogenase complex subunit C, SDHC) mutants (Ryu *et al*. 2016). Mitochondrial function was not impaired in the urolithin A treated animals, as exposure to carbonyl cyanide *p*-trifluoromethoxyphenylhydrazone (FCCP-an uncoupling agent), induced respiration over basal levels. Urolithin A extended the lifespan of young or old animals exposed to either paraquat, an inducer of ROS, or an antioxidant N-acetylcysteine. These findings suggest that its longevity effects are independent of ROS levels (Ryu *et al*. 2016).

In a recent publication, urolithin A supplementation reversed memory impairment through PINK-1, PDR-1, and DCT-1, suggesting that mitophagy is involved (Fang *et al*. 2019). Mitophagy diminished the insoluble Aβ_1-42_ and Aβ_1-40_ and prevented cognitive impairment of an APP/PS1 mouse model through microglial phagocytosis of extracellular Aβ plaques (Fang *et al*. 2019). It also abolished AD-related tau hyperphorphorylation in human neuronal cells and reversed memory impairment in a model that expresses Tau in *C. elegans* or in mice.

### Metformin treatment

The biguanide metformin, a drug that has been used for years as a first-line treatment for type 2 diabetes mellitus, promotes health, and extends lifespan in *C. elegans* (Onken and Driscoll 2010; Cabreiro *et al*. 2013; De Haes *et al*. 2014). The exact mode of action of metformin is not well understood. It is known to activate AMPK (Duca *et al*. 2015). Moreover, the transcription factor SKN-1/Nrf2, which regulates the transcription of antioxidants/cytoprotective genes, is required for the metformin-mediated increase in lifespan (Robida-Stubbs *et al*. 2012). Metformin has been found to extend lifespan in several model organisms (Bharath *et al*. 2020; Kulkarni *et al*. 2020; Lu *et al*. 2021). Restricted nuclear pore transit and upregulation of the ACAD10 were found to be required for metformin to extend lifespan (Wu *et al*. 2016). The expression of ACAD10 triggered by metformin requires SKN-1 activity (Wu *et al*. 2016), thus, it has been hypothesized that it limits MTORC1 activation by restraining the nucleo-cytosolic transport through the nuclear pore complex. It remains to be investigated whether the mode of action of metformin depends on the induction of bulk autophagy or some form of selective autophagy, such as mitophagy (Pietrocola and Kroemer 2017).

### Nicotinamide-adenine dinucleotide

NAD+ is an essential metabolite that participates in energy metabolism and many reduction-oxidation reactions (Wilson *et al*. 2023). NAD serves as a substrate for a series of NAD+ consuming enzymes, NADases, which include polyADP-ribose polymerases (PARPs) and the sirtuin family of deacetylases (SIRTs). NADases can directly regulate autophagy and mitochondrial quality control (Fang *et al*. 2014, 2016; Fang and Bohr 2017).

## Autophagy may also promote aging

More recently, there have been reports of examples of how autophagy can promote rather than inhibit senescent pathology. Sex-specific differences in autophagic function were found to influence longevity and to promote visceral aging (Ezcurra *et al*. 2018; Benedetto and Gems 2019). In this case, high levels of autophagy in the gut of hermaphrodites allows for production of more yolk and to maximize reproductive output. The DAF-2/IIR-driven and autophagy-mediated conversion of intestinal biomass into yolk eventually results in organ atrophy of the intestine and the accumulation of pseudocoelomic lipoprotein pools, as a form of senescent obesity (Ezcurra *et al*. 2018). In another example, global levels of autophagy were found to become dysfunctional with age and to be deleterious (Wilhelm *et al*. 2017). In these studies, post-reproductive inhibition of the VPS-34/BEC-1/EPG-8 autophagic nucleation complex in neurons, as well as its upstream regulators, strongly extended lifespan (Wilhelm *et al*. 2017). In contrast to previous studies that indicated positive roles of autophagy during aging, these data indicate that inhibition of early acting autophagy genes in aged worms results in improved neuronal integrity, and contributes to enhanced global health and increased longevity (Wilhelm *et al*. 2017). In these contexts, autophagy may be part of an example of antagonistic pleiotropy (Kirkwood 1977; Kirkwood and Austad 2000), where wild-type autophagy genes act beyond their “intended purpose” and are no longer under natural selection pressures.

Neuronal-specific RNAi depletion of early-acting autophagy genes extended lifespan, decreased polyQ aggregate number, and increased exopher biogenesis, an activity that requires ATG-16.2/ATG16L1 and its WD40 domain-related function (Yang *et al*. 2024). Neuronal exophers are large extracellular vesicles that are released from neurons to rid themselves of toxic protein aggregates (Melentijevic *et al*. 2017). A model has been proposed whereby inhibition of early acting autophagy genes promotes the formation of exophers, employing a noncanonical function of ATG-16.2/ATG16L1 WD40 domain that reduces neuronal protein aggregation and prolongs lifespan (Yang *et al*. 2024). Whether this ATG-16.2/ATG16L1 function represents a novel process of autophagy, is not known.

Autophagy was found to be detrimental for lifespan in conditions where mitochondrial membrane integrity is compromised, as in *sgk-1* or *rict-1* mutants (Zhou *et al*. 2021). Mutations in the serum/glucocorticoid-regulated kinase *sgk-1* or of the TORC2 component, rictor (*rict-1*), were shown to result in a short lifespan (Soukas *et al*. 2009), but have increased autophagy. Moreover, inactivation of autophagy genes by RNAi in the intestine specifically, suppressed the short-lived phenotype of *sgk-1* or *rict-1* mutants to wild-type levels (Soukas *et al*. 2009). An unbiased mass spectrometry analysis identified a group of regulators in the mitochondrial permeability transition pore (mPTP), including the voltage-dependent anion channel (VDAC), as interacting with SGK-1 (Zhou *et al*. 2021). SGK-1 directly phosphorylates the VDAC and regulates its degradation via the proteasome. Overexpression of *vdac-1* was reported to upregulate autophagy gene transcription and shorten lifespan, a phenotype that can be suppressed by the inhibition of autophagy (Zhou *et al*. 2021). This has led to the hypothesis that the transient or long-term opening of the mPTP is responsible for the short lifespan of *sgk-1* and *rict-1* mutants (Zhou *et al*. 2021). *vdac-1* overexpression suppresses the long-lived phenotypes of dietary-restricted (*eat-2* mutants), germline deficiency (*glp-1/Notch* mutants), or mitochondrial reduction (*nuo-6* and *frh-1* RNAi) (Zhou *et al*. 2021), in contrast to *daf-2/IIR* mutants which are still long lived despite *vdac-1* overexpression (Zhou *et al*. 2021). Thus, although autophagy has been demonstrated in many instances to be required for longevity, there are several instances where autophagy can be detrimental.

## Autophagy in stem cell homeostasis

In response to changes in the environment such as the availability of nutrients, and temperature, organisms delay development and/or reproductive capacity until favorable conditions resume (Hubbard and Schedl 2019). The *C. elegans* germ line is highly sensitive to food availability, and as such is a great model to investigate the role of autophagy in stem cell biology. In *C. elegans*, the reproductive organ consists of two U-shaped tubes that each contains approximately 1,000 germ cells, organized in a distal to proximal assembly line that contains the stem cell population in the distal end progenitor zone (Hubbard and Schedl 2019). As the cells move more proximally, they enter the meiotic program and will differentiate into sperm or oocytes. Each arm of the gonad has a cell population that proliferates during development, and which continues to divide after the animal reaches adulthood (Kimble and Crittenden 2005). For review, see Hubbard and Schedl (2019).

The decision of cells in the progenitor zone to proliferate is controlled by several signals that include the nutritional status of the animal and its age (Hubbard 2013; Hubbard and Schedl 2019). Several signaling pathways have been demonstrated to regulate the number of cells that account for the progenitor zone. These can affect either the cell cycle duration, or the decision of stem cells to proliferate vs differentiate (Hubbard and Schedl 2019). Germ cells have also been shown to be depleted with age, and the reproductive capacity of adult animals has been found to diminish with age (Luo and Murphy 2011; Pazdernik and Schedl 2013; Kocsisova *et al*. 2019; Tolkin and Hubbard 2021).

GLP-1/Notch signaling is required for the stem cell fate: in *glp-1/Notch* null mutant animals, germ cells begin to differentiate prematurely and enter meiosis in early larvae stages and spatially closer to the distal tip cell (Kimble and White 1981; Austin and Kimble 1987). In contrast, in *glp-1* gain-of-function mutants, the germline cells continue to divide, and they fail to enter meiosis, resulting in an overproliferation or Tumorous phenotype (Berry *et al*. 1997; Pepper *et al*. 2003; Hansen *et al*. 2004). Robust expansion of the cells in the proliferative zone is regulated by nutrient sensing pathways, such as the DAF-2/insulin IGF-1-like signaling pathway, and DAF-7/TGFβ signaling pathway (Korta and Hubbard 2010; Michaelson *et al*. 2010; Dalfo *et al*. 2012; Roy *et al*. 2016; Pekar *et al*. 2017). Larval progenitor zone cell accumulation also requires the activity of TORC1 as LET-363/mTOR, DAF-15/RAPTOR, the downstream effectors IFE-1/eIF4E and RSKS-1 p70 S6K are all required. DAF-18/PTEN, AAK-1/2 AMPK, and DAF-12/NHR function to establish the germline quiescence in dauer animals (Fukuyama *et al*. 2006, 2012; Narbonne and Roy 2006; Colella *et al*. 2016; Kadekar and Roy 2019; Tenen and Greenwald 2019). These signaling pathways bring about a complex set of signals and tissue-specific interactions that are not well understood.

The activity of autophagy genes was reported to be required for the normal accumulation of cells in the progenitor zone (Ames *et al*. 2017). Animals that carry a loss-of-function mutation in *bec-1/BECN1, atg-7/Atg7*, or *atg-18/Wipi2* genes, or RNAi knockdown (*atg-9, epg-1, bec-1*, *vps-34*, *epg-8, atg-12, cup-5*), were reported to display a reduction in the number of cells in the progenitor zone. *bec-1/BECN1, atg-7/Atg7*, or *atg-18/Wipi2* autophagy genes were found to promote cell cycle progression, and thus autophagy genes appear to be required to promote cell cycle progression in the adult (Ames *et al*. 2017). Detailed analysis of *bec-1/Becn1* mutants found that the number of germ cells replicating DNA or undergoing mitosis was decreased and that the cell cycle was delayed in an extension of the G2 phase (Ames *et al*. 2017). Intriguingly, BEC-1 was found to act in a nonautonomous manner to promote the cell cycle progression via the DAF-2 receptor (Ames *et al*. 2017). Similarly, *atg-18* also appears to act noncell-autonomously (Kosinski K and Meléndez A, unpublished results). One possible requirement for autophagy is as a source of nucleotides for germ line proliferation. Interestingly, RNST-2, a *C. elegans* T2 family endoribonuclease, was found to mediate autophagic degradation of ribosomal RNA in lysosomes (Liu *et al*. 2018). A genetic screen for lysosome-defective mutants identified mutations in *rnst-2*, which display autophagy-dependent accumulation of rRNA and ribosomal proteins within enlarged lysosomes (Liu *et al*. 2018). *rnst-2* loss-of-function mutants are defective in embryonic and larval development and are short-lived. Double mutants that combined the *rnst-2* loss of function with mutations in genes involved in pyrimidine biosynthesis resulted in complete embryonic lethality (Liu *et al*. 2018). It would be interesting to learn whether the degradation of ribosomal RNA in lysosomes is required for germ cell development.

A recent report found that autophagic recycling of nuclear material is an important cellular process that preserves nuclear architecture, restricts nucleolar size, and promotes longevity (Papandreou *et al*. 2023). Knockdown of the nuclear envelope anchor protein ANC-1/Nesprin-2 shortened the lifespan of long-lived *daf-2/IIR* mutants, to a similar extent to that of *bec-1/Becn1* depletion. Using a fibrillarin reporter strain FIB-1::GFP, *anc-1/nesprin-2* mutants were found to result in enlarged nucleoli and increased endogenous FIB-1::GFP protein levels, a phenotype similar to that of *bec-1* mutants (Papandreou *et al*. 2023). In contrast, *daf-2* knockdown resulted in reduced nucleolar size and decreased FIB-1::GFP (Papandreou *et al*. 2023). This report uncovers a germline immortality assurance mechanism that involves nucleolar degradation at the most proximal oocyte. Whether this has any connection with the mitotic phenotypes observed in autophagy mutants is not known.

## Autophagy in cell death

Autophagy has been implicated in promoting the clearance of apoptotic cells and in the process of cell death. It originally was thought to occur in apoptotic cells to promote the exposure of the phosphatidylserine, a step considered as part of the “eat me signal” that functions in signaling to the engulfing cell that will eventually degrade the apoptotic corpses (Qu *et al*. 2007). In *C. elegans* development, 131 somatic cells will die by programed, and this program is executed by a cascade of factors initiated by EGL-1, a BH3-only protein that binds to the antiapoptotic protein CED-9/BCL2, and activates CED-4/APAF-1 and CED-3 caspase activity (Hengartner and Horvitz 1994; Conradt 2009). Two evolutionarily conserved signaling pathways (e.g. one by CED-1, CED-6, and CED-7 and the other by CED-2, CED-5, and CED-12 in *C. elegans*) perceive a phosphatidylserine signal and activate a Rac GTPase (CED-10), leading to the reorganization of the actin cytoskeleton for phagocytosis (Reddien *et al*. 2001; Reddien and Horvitz 2004; Elliott and Ravichandran 2010). In the germ line, cells in the meiotic pachytene stage will undergo programmed cell death to become nurse cells that provide cytoplasmic components to the maturing oocytes (Gumienny *et al*. 1999). The corpses are removed by somatic sheath cells and the corpse clearance process is mediated by CED-1 and CED-5 parallel pathways.

Autophagy activity is required for the removal of cell corpses (Li *et al*. 2012; Huang *et al*. 2013; Wang *et al*. 2013; Jenzer *et al*. 2019; Peña-Ramos *et al*. 2022). Li *et al*. (2012) demonstrated a role for *lgg-1*, *atg-18* and *epg-5* in Q cell neuroblast corpse clearance in the L1 larval stage. Q neuroblasts at the left or right of the L1 larva generate two apoptotic cells (Q.aa and Q.pp) and three neurons by asymmetric cell division (Sulston and Horvitz 1977; Ou and Vale 2009; Ou *et al*. 2010). The neighboring hypodermal cell hyp7 engulfs and degrades the apoptotic Q cell. The autophagy proteins did not function in the initiation of apoptosis, but rather in the phagocyte to process the engulfed Q cell after their internalization and in phagosome maturation (Li *et al*. 2012).

In the germ line, Ruck *et al*. (2011) reported a role for *vps-34*, *bec-1/Becn1, unc-51/Ulk1,* or *atg-18/Wipi* in apoptotic germ cell corpse clearance. RNAi-depleted animals showed an increase in the number of apoptotic germ cell corpses, and the timing for cell clearance in *bec-1/Becn1* RNAi-depleted animals was longer than that of wild-type animals. TEM of *bec-1*-depleted animals, showed a fully engulfed apoptotic germ cell corpse, suggesting that the increase in apoptotic germ cell corpses was due to a delay in the cell corpse degradation and not an increase in germ cell death (Ruck *et al*. 2011). In contrast, Wang *et al*. (2013) found that mutations in *atg-3*, *lgg-1*, *atg-5*, *epg-1/Atg13*, *epg-4*, and *atg-2* did not affect the number of germ cell corpses, when compared with wild-type animals. The number of germ cell corpses was not increased in double-mutant combinations with the same autophagy genes and mutations in engulfment genes, such as *ced-1*, *ced-5*, or *ced-12*, as the double mutants were comparable to engulfment gene single mutants. In a third publication, *atg-13(bp414)*, *atg-9(bp564)*, *atg-4*.1*(bp501)*, and *atg-4*.2*(tm3948)* mutants showed higher levels of germ cell apoptosis than wild-type animals under physiological conditions (Min *et al*. 2019). In the report by Wang *et al*. (2013), they found that autophagy genes promote germ cell death following genotoxic stress (after γ ray or ENU treatment). They also found that autophagic activity can act to cooperate with caspases in the death of ventral cord neurons, in mutants with partially compromised function in *ced-3* (encoding the caspase) and *ced-4/APAF-1* (Wang *et al*. 2013). It is not clear why some genes in the pathway may have an effect while other genes do not. The role of autophagy in cell death and cell corpse removal may vary depending on the developmental context, the cell type, and the stage of the animal. It may also be that the increase in germ cell apoptotic nuclei observed in some autophagy mutants results from a mechanism different from autophagy. This has to be further examined. Both LGG-2/LC3 and LGG-1/GABARAP were found to be involved in phagocytosis of apoptotic corpses in an LC3-associated phagocytosis (LAP) process during embryonic development of *C. elegans* (Jenzer *et al*. 2019). LGG-1 was found to act in apoptotic cells for the surface exposure of phosphatidylserine and LGG-2 was found to mediate the fusion between phagosome and lysosome to promote the degradation of apoptotic cells (Jenzer *et al*. 2019). More recently, Peña-Ramos *et al*. (2022) employed time-lapse fluorescence microscopy to show that canonical double-membrane autophagosomes are recruited to phagosomes to promote degradation of cell corpses by controlling the acidification of the phagosome in the embryo. Peña-Ramos describes a novel interaction between phagosomes and autophagosomes in the degradation of apoptotic cell corpses. In mammalian cells, lipidated LC3 molecules have been reported to label LAP vesicles, which are single-membrane vesicles (Sanjuan *et al*. 2007). LAP vesicles fuse to phagosomes and facilitate the degradation of apoptotic cells in mice (Martinez *et al*. 2011; Green *et al*. 2016) and require the function of autophagosome biogenesis genes, but not ULK1, ATG13, or ATG14. However, LGG-1 and LGG-2 define subpopulations of autophagosomes (LGG-1 positive only, LGG-2 positive only, and LGG-1 and LGG-2 positive) that all contribute to the degradation of apoptotic cell corpses. In the process of phagosome acidification, mCherry -LGG-1 and -LGG-2 reporters were found inside the phagosomal lumen, and this process required the autophagosome biogenesis genes *atg-13* and *epg-8/Atg14*, genes that act in the canonical autophagy pathway and have not been found to be required for LAP (Peña-Ramos *et al*. 2022). In addition, ATG-9, the only membrane spanning autophagosomal protein was found to be present in phagosomal lumen, thus autophagosomes act in addition to lysosomes and endosomes in the acidification of phagosomes (Peña-Ramos *et al*. 2022). This report has worked to establish that in *C. elegans*, autophagosomes can fuse with the phagosome and are required as part of the maturation process of phagosomes (Peña-Ramos *et al*. 2022).

Studies have found that lysosomes go through a process of reformation from autolysosomes, referred to as autophagic lysosome reformation (ALR) (Gan *et al*. 2019). In *C. elegans*, lysosomal reformation occurs following phagolysosomal digestion of cell corpses in embryos, referred to as phagocytic lysosome reformation (PLR). SLC-36.1, the *C. elegans* ortholog of mammalian neutral amino acid transporters SLC36A1-4 (PAT1-4) is an essential regulator of PLR, and together with PPK3, the *C. elegans* PIKfyve ortholog, they are required for PLR (Gan *et al*. 2019). In the adult hypodermis, where no cell death occurs, SLC-36 and PPK-3 were found to also play a role in ALR (Gan *et al*. 2019).

Excessive autophagy has also been associated with necrotic cell death (Samara *et al*. 2008). Necrotic cell death can be suppressed by inactivation of autophagy genes or impairment of autophagy by pharmacological treatment (Samara *et al*. 2008). Necrosis in *C. elegans* is mostly defined by its physiological characteristics, which include plasma membrane whorls, cytoplasmic swelling, the formation of vacuoles, and nuclear membrane disintegration with a lack of chromatin condensation (Hall *et al*. 1997). The role of autophagy in necrosis was observed in the degeneration of touch cell neurons in animals that carry gain-of-function mutations in *mec-4, mec-4d* (Driscoll and Chalfie 1991; Toth *et al*. 2007; Samara and Tavernarakis 2008). Loss-of-function mutations in *unc-51* or *bec-1*, or RNAi depletion of *lgg-1* suppressed the necrotic-like degeneration. Excessive autophagy induced by the gain-of-function mutation in *mec-4, mec-4d,* supported the idea that excessive levels of autophagy play a role in cell death by necrosis (Toth *et al*. 2007; Samara *et al*. 2008). In a recent report on the death of mechanosensory OLQ neurons and uv1 neuroendocrine cells, which die in a *pnc-1* mutant background as a result of excess nicotinamide (NAM) (Huang and Hanna-Rose 2006). PNC-1 encodes nicotinamidase and catalyzes the first step in the recycling of NAM to NAD^+^ (Huang and Hanna-Rose 2006; van der Horst *et al*. 2007). Even if the phenotypes of the dying cells by necrosis are similar, *unc-51* and *bec-1* were required for OLQ necrosis but had no role in uv1 necrosis (Reza *et al*. 2022). Thus, there are different types of necrosis. Why this is the case is not known.

## Autophagy in innate immunity

*C. elegans* in their natural habitat are exposed to times of starvation and a wide variety of microbes that can be food, commensals, or pathogens (Schulenburg and Felix 2017; Zhang *et al*. 2017a). To defend against infection, *C. elegans* rely on innate immunity, rather than an adaptive immune system (Ermolaeva and Schumacher 2014; Kim and Ewbank 2018). Several recent studies have begun to elucidate the role of autophagy and proteostasis in response to pathogen infection (Kuo *et al*. 2018; Gallotta *et al*. 2020; Martineau *et al*. 2021). It is interesting to consider the connection of the signaling pathways that respond to nutrient deprivation, and those that act to control aging and how they also regulate the immune response. Disturbances that include starvation, damage of organelles, hypoxia, heat stress, and pathogen infection all impinge on the autophagic process, and autophagy as part of the proteostatic network, is important for the immune response.

## Autophagy is required for resistance to *Salmonella enterica*

A first role for autophagy in the defense to infection in *C. elegans* was documented by exposing animals to the gram-negative bacterium *Salmonella enterica Serovar Typhimurium* (Jia *et al*. 2009). *S. enterica* establishes a persistent infection in the lumen of the *C. elegans* intestine, thereby inflicting damage, and ultimately killing the host animal (Aballay *et al*. 2000, 2003). Mutations in *daf-2/IIR,* and in the class I phosphatidylinositol 3-kinase *age-1*, confer resistance to *S. enterica* infection (Garsin *et al*. 2003; Kurz and Tan 2004). Autophagy genes were required to inhibit the persistence of the pathogen and its replication in *daf-2/IIR* mutants (Jia *et al*. 2009). In animals that overexpress DAF-16/FOXO, it was noted that an increase in GFP::LGG-1 positive foci occurs in seam cells, a reporter for autophagosomes (Meléndez *et al*. 2003). In addition, the overexpression of DAF-16/FOXO required the activity of autophagy genes to provide pathogen resistance (Jia *et al*. 2009). RNAi depletion of *bec-1/Becn1* or *lgg-1/Atg8* resulted in the accumulation of intracellular bacteria and an expansion in the bacterial population in the intestinal lumen, demonstrating that autophagy genes are essential in host defense and mediate pathogen resistance (Jia *et al*. 2009). Intestinal-specific expression of the autophagy gene *bec-1/Becn1* was found to be essential for host defense against Salmonella infection, demonstrating a cell autonomous requirement (Curt *et al*. 2014). This may be an example of xenophagy, a phenomenon first described in mammals (McEwan 2017), which involves recognition and ubiquitinylation events that mark intracellular pathogens for autophagosomal degradation (Andrade *et al*. 2006; Deretic 2006; Ling *et al*. 2006; Levine and Deretic 2007). However, this is not clear, as *S. enterica* does not appear to enter the cell (Kuo *et al*. 2018). Infection with pathogenic *S. enterica* was also shown to induce autophagy by inactivating the target of rapamycin (mTOR) (Ma *et al*. 2021). Although the mechanisms involving autophagy in limiting bacterial proliferation and reducing the load of bacteria in the lumen are not well understood, clearly autophagy is required for increased survival against Salmonella infection.

## Autophagy in necrosis after *Pseudomonas aeruginosa* infection

Autophagy was found to play an important role in host defense after the infection with *Pseudomonas aeruginosa* (Zou *et al*. 2014). Infection with *P. aeruginosa* (PA14) activates the extracellular signal-regulated kinase (ERK) pathway by upregulating the expression of the epidermal growth factor (EGF) ligand gene *lin-3*. The ERK pathway contributes to the activation of autophagy through its substrate CDC-48.2 (Zou *et al*. 2014). CDC-48.2 encodes a homolog of mammalian p97/VCP and yeast Cdc48, type II AAA ATPases. A significant increase in the GFP::LGG-1 reporter and lipidation of LGG-1 detected by Western blot were noted after infection with PA14 (Zou *et al*. 2014). Interestingly, heat-killed bacteria did not induce autophagy, suggesting that the pathogenicity of *P. aeruginosa* activates autophagy. Systemic RNAi of *mpk-1* significantly suppressed PA14-induced autophagy, whereas RNAi-mediated depletion of DAF-16/FOXO in the intestine had no effect on autophagy induction, even if it significantly enhanced susceptibility of PA14 infection (Zou *et al*. 2014). Mutations in the ERK pathway, such as *let-60/ras, lin-45/raf, mec-2, or mpk-1* enhanced the sensitivity of animals to PA14 infection, and in contrast, the gain-of-function mutation in *let-60* resulted in enhanced resistance to PA14 infection (Zou *et al*. 2014). Autophagy appeared to inhibit necrosis, and in turn, enhance the survival of infected animals. In these experiments, inhibition of autophagy did not influence the intestinal colony counts of *P. aeruginosa* or *Staphylococcus aureus*, suggesting that the primary protective role of autophagy was not to directly eliminate the pathogen, but to ameliorate necrosis by a not well-understood mechanism (Zou *et al*. 2014).

*C. elegans* infection by *P. aeruginosa* induces an iron-related mitophagy. Pyoverdine, a siderophore produced by *P. aeruginosa*, is required for pathogenesis in *C. elegans* (Kirienko *et al*. 2015). Siderophores are soluble extracellular molecules secreted by invasive microorganisms to bind and scavenge iron storage proteins from the host (Cassat and Skaar 2013; Kirienko *et al*. 2013). Pyoverdine binds iron, and provides iron to the pathogen, a key virulence factor (Meyer *et al*. 1996). Pyoverdine translocates to the host cell, binds, and extracts iron, as it damages the host mitochondria (Kang *et al*. 2018). Pyoverdine can kill *C. elegans* in the absence of bacterial pathogen (Kirienko *et al*. 2015). RNAi depletion of autophagy genes *bec-1/Becn1, lgg-1* or the mitophagy regulators *pink-1/PINK1* and *pdr-1/PARKIN* sensitized *C. elegans* to *P. aeruginosa* infection (Kirienko *et al*. 2015). Thus, mitophagy induced by iron chelation eliminates damaged mitochondria, to preserve energy homeostasis in this type of pathogen infection. It has been proposed that *C. elegans* surveils neuronal mitochondrial dynamics, to coordinate a systemic mitochondrial unfolded protein (UPRmt) response with mitochondrial connectivity, and to optimize survival under bacterial infection (Chen *et al*. 2021a). Activation of the mitochondrial UPR and mitochondrial fragmentation improved resistance to pathogenic P14 infection (Chen *et al*. 2021a).

In a recent publication, the CF18 strain of *P. aeruginosa* was found to cause a severe but reversible developmental delay that was shown to be caused by the induction of ROS and mitochondrial dysfunction (Mirza *et al*. 2023). In the response to infection, larvae upregulated mitophagy, antimicrobial, and detoxification genes, while the UPRmt response genes were repressed. The recovery and survival of mitophagy mutants *pink-1*, *dct-1*, and *pdr-1/Parkin* was significantly decreased, when compared with wild-type animals exposed to C18, suggesting that in this case mitophagy is also involved.

## Infection by *S. aureus* regulates HLH-30/TFEB

Besides its role during nutritional stress, HLH-30/TFEB plays an important role in the immune response, for example in the response to the extracellular bacterium *S. aureus* (Visvikis *et al*. 2014). *S. aureus* infection of *C. elegans* by the oral route entails colonization of the intestinal lumen, intestinal epithelial cell destruction, and death to the animal within 48 h (Sifri *et al*. 2003; Irazoqui *et al*. 2010). HLH-30/TFEB is activated early during infection and controls the expression of the transcriptional immune response to infection (Visvikis *et al*. 2014). HLH-30 acts in a positive feedback loop during infection and orchestrates the regulation of components of several signaling pathways implicated in host defense, including JNK, p38MAPK, INS-18/INS, and TGF-β. HLH-30/TFEB also controls the expression of antimicrobial genes, such as autophagy genes, lysozymes, and C-type lectins, all necessary for host defense. RNAi-mediated depletion of *lgg-1/Atg8, unc-51/Atg1,* or *vps-34/VPS34/pik3c3*, resulted in impaired survival of *S. aureus* infection (Irazoqui *et al*. 2010). *S. aureus* infection induces the translocation of HLH-30/TFEB to the nucleus, similar to what has been shown in response to nutritional stress (Lapierre *et al*. 2013; O'Rourke and Ruvkun 2013), and mutants lacking HLH-30/TFEB exhibit strong host defense defects. Mechanistically, HLH-30 drives most of the transcriptional host response, and both HLH-30/TFEB-regulated antibacterial and autophagy genes are required for host tolerance of infection (Visvikis *et al*. 2014).

The orphan nuclear receptor NHR-42 is an important negative regulator of host infection resistance, and functions at the intestinal epithelium, the site of infection by *S. aureus* (Goswamy *et al*. 2023). NHR-42 represses innate immunity and promotes lipid loss downstream of HLH-3/TFEB (Goswamy *et al*. 2023). Transcriptional profiles of *nhr-42* mutants showed activation of an antimicrobial signature, of which *abf-2, cnc2*, and *lec-11* appeared to be important for the enhanced survival of infection of *nhr-42* mutants (Goswamy *et al*. 2023). Curiously, *abf-2* is repressed by *nhr-42* in the pharynx, whereas *nhr-42* is expressed throughout the body (Goswamy *et al*. 2023). These results advance our understanding of the mechanisms by which the microphthalmia-TFE (MiT) family of transcription factors promote host defense and suggest that HLH-30/TFEB and TFE3 may similarly promote host defense via NHR-42-homologous nuclear receptors in mammals (Goswamy *et al*. 2023). Since autophagy requires membrane rearrangements and is involved in damaged organelle recycling, lipids may serve as a source of energy in host cytoprotection (Hou and Taubert 2012). In the absence of lipid mobilization, *nhr-42* mutants might engage in autophagy to obtain energy for host defense from alternative sources (Hou and Taubert 2012).

HLH-30/TFEB integrates organismal stress, the response to starvation, and pathogen recognition as it coordinates host responses to all these different stresses (Lapierre *et al*. 2013; O'Rourke and Ruvkun 2013; Settembre *et al*. 2013a; Visvikis *et al*. 2014; Raben and Puertollano 2016). How HLH-30/TFEB integrates this information to produce specific responses to the different stresses and what factors may contribute to HLH-30/TFEB specificity are not known. We should note that the transcriptional response after *S. aureus* infection was distinct from the transcriptional response induced by nutritional deprivation, defining an infection-specific transcriptional signature (Wani *et al*. 2021). Thus, it will be important to understand how HLH-30/TFEB integrates the information to produce a specific response.

### *Nematocida parissii* and Orsay virus infection induces autophagy

*C. elegans* can be infected by microsporidia, an intracellular pathogen that can infect a wide variety of animal hosts, including humans (Didier 2005; Williams 2009; Troemel 2011). *N. parisii* are natural intracellular parasites that were isolated from wild-caught animals (Hodgkin and Partridge 2008; Troemel *et al*. 2008; Zhang *et al*. 2016). *N. parisii* employs an infection apparatus, which delivers the parasite directly into the host cell, where it replicates intracellularly. Autophagy and ubiquitination play a role in controlling *N. parisii* intestinal infection in *C. elegans* (Bakowski *et al*. 2014). Another natural intracellular pathogen of *C. elegans* is the Orsay virus, which was found originally in wild-isolates of *C. elegans*. The Orsay virus is a positive-strand RNA virus of the family *Nodaviridae* (Félix *et al.* 2011), and similar to *N. parissii*, it replicates inside *C. elegans* intestinal cells. Infection with *N. parissi* or with the *Orsay virus* upregulates the expression of genes involved in ubiquitylation (Bakowski *et al*. 2014). Transcriptional profiling of genes induced under infection with *N. parissii* identified several genes encoding components of the Skp1-Cul1-F-box protein (SCF) E3 ubiquitin ligases, including *cul-6*, *skr-3*, or *skr-5* (Bakowski *et al*. 2014). A significant increase in pathogen load was observed in animals RNAi depleted for the components of the SCF E3 ubiquitin ligase, suggesting that the E3 ligase components, together with ubiquitin-mediated proteolysis, limit growth of *N. parissii* during infection (Bakowski *et al*. 2014). A similar induction of the SCF E3 ligase components was observed after transcriptional profiling during infection with Orsay virus. Restriction of *N. parissii* growth appeared to depend on both the proteasome and the autophagy pathway (Bakowski *et al*. 2014). During the parasite replication phase, LGG-1/Atg8 and ubiquitin co-localize to the *N. parisii* within the intestinal cells and RNAi depletion of *lgg-1/Atg8* or *sqst-1/p62* resulted in an increase in pathogen load. In addition, activation of autophagy by RNAi depletion of the nutrient sensor *let-363/mTOR*, increased the recognition driven by LGG-1/Atg8 targeting and resulted in a reduced pathogen load (Bakowski *et al*. 2014). Since the increase in pathogen load after RNAi of autophagy genes was relatively small, this result has been interpreted such that *N. parissii* may actively suppress autophagy (Kuo *et al*. 2018). Treatment with the DNA synthesis inhibitor fluorodeoxyruidine (FuDR) or the antimicrosporidia drug fumagillin resulted in an increase in the efficiency of ubiquitin targeting to parasite cells, suggesting that ubiquitination and autophagy both control intestinal infection with *N. parissii*, an example of xenophagy (Kuo *et al*. 2018).

### Pore-formin-toxins from *Bacillus thuringiensis*

Another example for the role of autophagy in host defense is in the response to pore-forming-toxins (PFTs), which damage host cellular membranes (Verma *et al*. 2021). Upregulation of the hypoxia pathway was found to be required for animals to be resistant to PFTs (Bellier *et al*. 2009), thus low oxygen and the hypoxia pathway are important for the response to PFTs and the protection of cells that are directly attacked by the PFTs. A recent report found that the PFTs Cry5B and Cry21A are produced by the extracellular gram-positive bacterium *B. thuringiensis* and induce autophagy via HLH-30/TFEB (Chen *et al*. 2017a). In animals fed with *Escherichia coli* expressing the Cry5B toxin, an increase in autophagic vesicles was detected by electron microscopy and an increase in positive foci with the GFP::LGG-1 reporter was noted, which colocalized with labeled Cry5B proteins inside intestinal cells (Chen *et al*. 2017a). The induction of autophagy was cell autonomous and HLH-30/TFEB dependent. RNAi depletion of several autophagy genes, including *bec-1/Becn1, atg-4.1*, *atg-4.2*, *lgg-1*, *lgg-2*, *lgg-3*, and *atg-18/Wipi2* all decreased the survival of infected animals with *E. coli* that expressed Cry5B. In addition, autophagy genes were required for the repair of membranes after Cry5B damage. Thus, PFTs elicits a cell autonomous induction of autophagy that controls the tolerance to PFT intoxication and xenophagic degradation of PFTs, as well as repair of the damaged membrane (Chen *et al*. 2017a). In this case, PFTs alone can elicit the response, and autophagy serves as an innate immune mechanism against bacterial infection.

## Other processes that employ part of the autophagy machinery

A subset of the autophagy machinery proteins are required for LC3/ATG8 lipidation to single-membrane vesicles, a process known as conjugation of ATG8 to single membranes (Durgan *et al*. 2021), which function in pathways that include the LC3-associated phagocytosis (LAP), and the LC3-associated endocytosis (LANDO). Both LAP and LANDO rely on some of the members of the autophagy pathway to allow for efficient degradation of the cargo. LAP vesicles are single-membrane vesicles, instead of the double-membrane canonical autophagosomes (Sanjuan *et al*. 2007; Nakatogawa 2020). Both LAP and LANDO require the ultraviolet radiation resistance-associated gene protein (UVRAG) and Rubicon (Itakura *et al*. 2008; Heckmann *et al*. 2017; Heckmann and Green 2019; Pena-Martinez *et al*. 2022). UVRAG and Rubicon take the place of AMBRA1 and ATG14L in the autophagy VPS-34/PI3KC3 complex. AMBRA and ATG14L are both dispensable for LAP or LANDO activation, as is also the autophagy preinitiation complex containing FIP200/ULK1 in mammals (Heckmann *et al*. 2019; Heckmann and Green 2019). Although UVRAG and Rubicon orthologs have been identified in *C. elegans* based on protein sequence similarity (Table 1), no ortholog for Ambra has been identified in *C. elegans*. Another difference between autophagy and LAP is the requirement for reactive oxygen species (ROS) production in LAP (Heckmann *et al*. 2017; Heckmann and Green 2019; Boada-Romero *et al*. 2020). The cargo engulfed by autophagosomes is derived from intracellular sources, whereas LAP and LANDO cargo originate from the extracellular environment (Pena-Martinez *et al*. 2022).

Recent evidence has found that the clearance of the second polar body in *C. elegans* provides a strong model to study LAP (Fazeli *et al*. 2016, 2018). During meiosis, the oocyte expels the polar bodies to avoid lethal polyploidy (Fazeli *et al*. 2016, 2018). Polar bodies lose membrane integrity and expose phosphatidylserine. Polar body signaling recruits engulfment receptors to the plasma membrane of embryonic blastomeres using VPS-34/PI3K, the RAB-5 GTPase and the sorting nexin SNX-6 (Fazeli *et al*. 2018). LC3 lipidation is required for degradation of the corpse membrane after lysosome fusion. The polar body phagolysosome vesiculates in an mTOR- and ARL-8-dependent manner, which ensures its timely degradation (Fazeli *et al*. 2018).

Undifferentiated embryos degrade the midbody by LAP, a process independent of canonical autophagy (Fazeli *et al*. 2016). The midbody is released and phagocytosed before it undergoes phagosome degradation (Fazeli *et al*. 2016). This process requires the RAB-5 GTPase to localize the class III phosphoinositide 3-kinase VPS-34 complex at the cortex and the autophagy proteins BEC-1/BECN1, and LGG-1/2 are required, whereas UNC-51/ULK and EPG-8/ATG14 are not required for degradation (Fazeli *et al*. 2016). In polar body and midbody clearance, LGG-1/2 facilitates the lysosomal degradation of large, membrane-wrapped cargo. How LGG-1/2 is required to disrupt the inner membranes is not well understood.

In another process implicating autophagy proteins, the GABARAP ortholog LGG-1 controls the size of the nucleolus, which is a key hub for ribosomal assembly and an important lifespan determinant (Kumar *et al*. 2022). Silencing the conserved nuclear export receptor Exportin 1/XPO-1 led to marked reduction in global translation, together with a decrease in nucleolar size and lower levels of the nucleolar rRNA methyl transferase fibrillarin/FIB-1 (at the transcriptional and translational level) (Kumar *et al*. 2022). A connection with protein degradation and ribosome protein surveillance was established, as the ribosomal large subunit protein RPL-11 was found to be a target of LGG-1-mediated degradation.

## Conclusion

Over the past few decades, great strides have been made on the characterization of the autophagy machinery and its roles in *C. elegans* development, maintaining cellular homeostasis, and longevity. These studies highlight the unique strengths of *C. elegans* as a model system, where genetic screens have allowed for the identification of previously unknown, conserved autophagy genes and to study their functions in animal physiology, metabolism, stress, and in longevity. *C. elegans* continues to provide an excellent tool for the discovery of novel genes and critical roles for autophagy genes in synapse formation, metabolism, cellular homeostasis, clearance of apoptotic corpses, clearance of toxic aggregate-prone proteins, innate immunity, and longevity. In addition, autophagy is often found to be disrupted in age-related disorders such as cancer, diabetes, and neurodegenerative diseases, and to be required for pro-longevity regimens. Research in *C. elegans* has clearly contributed to a better understanding of the molecular mechanisms underlying these processes and the physiological roles of autophagy at the level of a whole organism, including nonautonomous effects and the communication between different tissues.

Since their initial discovery, many ATG proteins have been ascribed functions beyond that of autophagosome formation or ATG8-lipid conjugation. For example, BEC-1/Becn1, a component of the VPS-34 complex, regulates the nucleation step of autophagy (Kihara *et al*. 2001), but it has also been implicated in several nonautophagy functions, including endocytosis and retromer function in *C. elegans* (Ruck *et al*. 2011). Thus, future studies that examine the role of autophagy should rely on null mutations of more than one autophagy gene, preferably investigating genes with early and late functions in the pathway and employing inducible systems that can differentiate between canonical and noncanonical autophagy phenotypes.

The pleiotropic nature of autophagy genes requires the use of conditional knockout experiments, which can determine the spatial and temporal requirements for a gene in a specific context and how this context-dependent contribution of a gene affects a particular phenotype. Tissue-specific RNAi knockdown (Qadota *et al*. 2007; Zhuang and Hunter 2011; Zou *et al*. 2019) or recombinase-mediated conditional expression, such as employing Cre/lox, FLP/FRT, or Flexon experiments should be considered (Davis *et al*. 2008; Voutev and Hubbard 2008; Hubbard 2014; Muñoz-Jiménez *et al.* 2017; Shaffer and Greenwald 2022). Auxin-inducible degradation technology (Zhang *et al*. 2015b; Ashley *et al*. 2021; Negishi *et al*. 2022) will also be very helpful in the future as these can also allow for the high efficiency and rapid depletion of targets, as well as the reversibility of the system. Looking forward, it will be powerful to elucidate how the common components interface between the different canonical and noncanonical autophagic gene functions and how these affect development, cellular homeostasis and physiology.

*C. elegans* is an excellent model organism to study the cellular processes required for longevity and we expect that we will continue to build on the knowledge of the pathways involved, how they require autophagy for longevity, the tissues involved and the cellular processes that interface with autophagy. This will also help elucidate mechanisms of human disease. In the past few years, research in *C. elegans* has uncovered new layers of complexity for how genes function to protect a multicellular organism against stress, and control longevity. It may be difficult to differentiate between canonical autophagy and selective forms of autophagy because of the lack of effective methods to positively or negatively manipulate the selective processes without also affecting the canonical autophagy process. How different forms of selective autophagy cooperate to maintain cellular and organismal homeostasis, and the interplay between these pathways in pro-longevity regimens are interesting avenues of research to be investigated in the future. The advantage of the genetic tractability of *C. elegans* and its evolutionary conservation, will continue to elucidate the mechanisms involved and highlight the use of *C. elegans* as a model system.
